# Causal association of obesity with epigenetic aging and telomere length: a bidirectional mendelian randomization study

**DOI:** 10.1186/s12944-024-02042-y

**Published:** 2024-03-12

**Authors:** Jixin Li, Wenru Wang, Zhenyu Yang, Linjie Qiu, Yan Ren, Dongling Wang, Meijie Li, Wenjie Li, Feng Gao, Jin Zhang

**Affiliations:** 1 Chinese Academy of Traditional Chinese Medicine, Xiyuan Hospital, Beijing, China; 2 Heilongjiang University Of Chinese Medicine, Harbin, China

**Keywords:** Obesity, Epigenetic age, Telomere, Mendelian randomization, Genome-wide association study

## Abstract

**Background:**

In observational studies, there exists an association between obesity and epigenetic age as well as telomere length. However, varying and partially conflicting outcomes have notably arisen from distinct studies on this topic. In the present study, two-way Mendelian randomization was used to identify potential causal associations between obesity and epigenetic age and telomeres.

**Methods:**

A genome-wide association study was conducted using data from individuals of European ancestry to investigate bidirectional Mendelian randomization (MR) regarding the causal relationships between obesity, as indicated by three obesity indicators (body mass index or BMI, waist circumference adjusted for BMI or WCadjBMI, and waist-to-hip ratio adjusted for BMI or WHRadjBMI), and four epigenetic age measures (HannumAge, HorvathAge, GrimAge, PhenoAge), as well as telomere length. To assess these causal associations, various statistical methods were employed, including Inverse Variance Weighted (IVW), Weighted Median, MR Egger, Weighted Mode, and Simple Mode. To address the issue of multiple testing, we applied the Bonferroni correction. These methods were used to determine whether there is a causal link between obesity and epigenetic age, as well as telomere length, and to explore potential bidirectional relationships. Forest plots and scatter plots were generated to show causal associations between exposures and outcomes. For a comprehensive visualization of the results, leave-one-out sensitivity analysis plots, individual SNP-based forest plots for MR analysis, and funnel plots were included in the presentation of the results.

**Results:**

A strong causal association was identified between obesity and accelerated HannumAge, GrimAge, PhenoAge and telomere length shrinkage. The causal relationship between WCadjBMI and PhenoAge acceleration (OR: 2.099, 95%CI: 1.248—3.531, *p* = 0.005) was the strongest among them. However, only the *p*-values for the causal associations of obesity with GrimAge, PhenoAge, and telomere length met the criteria after correction using the Bonferroni multiple test. In the reverse MR analysis, there were statistically significant causal associations between HorvathAge, PhenoAge and GrimAge and BMI, but these associations exhibited lower effect sizes, as indicated by their Odds Ratios (ORs). Notably, sensitivity analysis revealed the robustness of the study results.

**Conclusions:**

The present findings reveal a causal relationship between obesity and the acceleration of epigenetic aging as well as the reduction of telomere length, offering valuable insights for further scientific investigations aimed at developing strategies to mitigate the aging process in humans.

**Supplementary Information:**

The online version contains supplementary material available at 10.1186/s12944-024-02042-y.

## Introduction

The global obesity issue is now an indisputable fact, with the prevalence of obesity having doubled in over 73 countries since 1980 [1]. With the exception of Saharan Africa and a few countries with low obesity rates (Sri Lanka, Indonesia, etc.), all regions of the world have a severe prevalence of obesity [2]. Obesity is a key risk factor for type 2 diabetes, cardiovascular disease, non-alcoholic fatty liver disease, hypertension, chronic kidney disease, polycystic ovary syndrome, depression, sleep apnea, and several tumors, significantly elevating patient mortality [3–5]. It has been estimated that healthcare costs for obese individuals are 30% higher than those of normal weight [2]. Moreover, the World Obesity Federation, along with the American and Canadian Medical Associations, has officially recognized obesity as a chronic and progressive disease. This distinction underscores that obesity differs significantly from other risk factors associated with diseases [6]. Hence, our continued investigation into the epidemiology of obesity aims to elucidate the connection between obesity and various comorbidities. This endeavor holds the potential to offer insights for the prevention and treatment of both obesity and its associated conditions.

With the development of epigenetics, many studies have reported that obesity is inextricably linked to accelerated epigenetic age [7–9]. Moreover, epigenetic age acceleration has been associated with various metabolic diseases [10], cardiovascular diseases [11, 12]. cancers [10, 13] and other adverse outcomes [14]. As is widely recognized, chronological age, measured in terms of time, does not precisely capture the actual aging status of the human body, including its tissues and organs. Individual aging is influenced by a complex interplay of confounding and genetic factors [15]. Consequently, there has been rapid development in the field of biological age measurement methods, such as the epigenetic clock and telomere depletion, which aim to provide a more accurate assessment of an individual's aging process [16, 17]. The epigenetic clock is an accurate marker that responds to aging obtained by measuring DNA methylation (DNAm) at Cytosine-phosphate-guanine (CpG) loci in different groups [18]. The first generation of epigenetic aging clocks include HannumAge [19] and HorvathAge [20]. HannumAge was obtained by training 71 CpGs loci in blood, while HorvathAge was obtained through training on 353 CpGs age-related loci found in human cell and tissue species. The second generation of epigenetic aging clocks include PhenoAge [10] and GrimAge [21]. PhenoAge is based on data from 513 CpGs that are associated with mortality risk, along with nine clinical biomarkers, including parameters like white serum glucose, C-reactive protein, mean corpuscular volume, albumin, creatinine, and others. On the other hand, GrimAge is derived from 1030 CpGs associated with the smoking factor and incorporates data from seven plasma proteins, such as adrenomedullin and growth differentiation factor 15, as key components. Hence, variations in the prominence of the four epigenetic age metrics stem from disparities in their training methodologies [22]: Notably, HorvathAge stands out as the initial age estimation tool with broad applicability across various tissues and organs. In contrast, HannumAge exhibits enhanced accuracy in predicting age when applied to adult blood samples. The limited correlation between these two primary genetic age proxies concerning clinical characteristics such as lipids, blood pressure, and glucose is compensated for by the utilization of the second epigenetic aging clocks markers. Additionally, PhenoAge excels in predicting mortality over extended periods (10–20 years), while GrimAge demonstrates a distinct advantage in leveraging lifestyle indicators for age prediction. Telomeres are located at the ends of chromosomes and serve to prevent chromosome degradation [23]. Telomere length shortens with cell division, thereby correlating with cellular senescence. Studies have demonstrated [24, 25] that telomere length is strongly associated with cardiovascular disease, cancer and the upper limit of the human life span.

At present, there are numerous studies on the correlation of obesity with biological age and telomeres. Kresovich et al. [26] discovered that body mass index (BMI) and waist circumference (WC) were significantly associated with high values of four epigenetic clocks through a cross-sectional analysis of 2758 women. However, Foster et al. [27] in a cross-sectional study involving a sample of 290 cases, reported findings that did not align with the results of Kresovich et al. [26]. Foster's study found no significant associations between BMI and WC with higher HannumAge in women. Additionally, neither BMI nor WC was found to be associated with GrimAge in their research. This limitation might be attributed to the constraints of their study; Foster et al.'s research suffered from a small sample size and was susceptible to inherent biases. Furthermore, the aforementioned studies have several limitations in that they primarily assessed correlations but could not establish causation. Additionally, they may not have completely controlled for the influence of confounding factors. Hence, the observed disparity in their outcomes could be attributed to the aforementioned factors. Loh et al. [28] found a significant negative correlation between BMI and telomere length through Mendelian randomization (MR) analysis; however, when it came to waist-to-hip ratio (WHR), the causal association with telomere length was not statistically significant. Notably, there existed a failure to investigate the inverse relationship in that study, with WC and other biological age indicators not being taken into account. A unidirectional Mendelian randomization analysis of BMI and WC with GrimAge and PhenoAge by Kong et al. [29] confirmed a positive facilitating effect, but the reverse relationship has not been explored and the inclusion of biological markers of age is incomplete. Investigating the inherent connection between obesity and biological age markers is instrumental in elucidating the mechanisms through which obesity contributes to the development of other diseases. For instance, obesity has been shown to influence the methylation of genes involved in lipid metabolism, such as ABCG1 and NOD2 [30]. This knowledge can provide insights into the prevention and management of conditions like dyslipidemia. Thus, addressing the academic gap of standardizing and comprehensively measuring the causal association between obesity and biological age is currently relevant, yet it lacks systematic research.

**Fig. 1:**
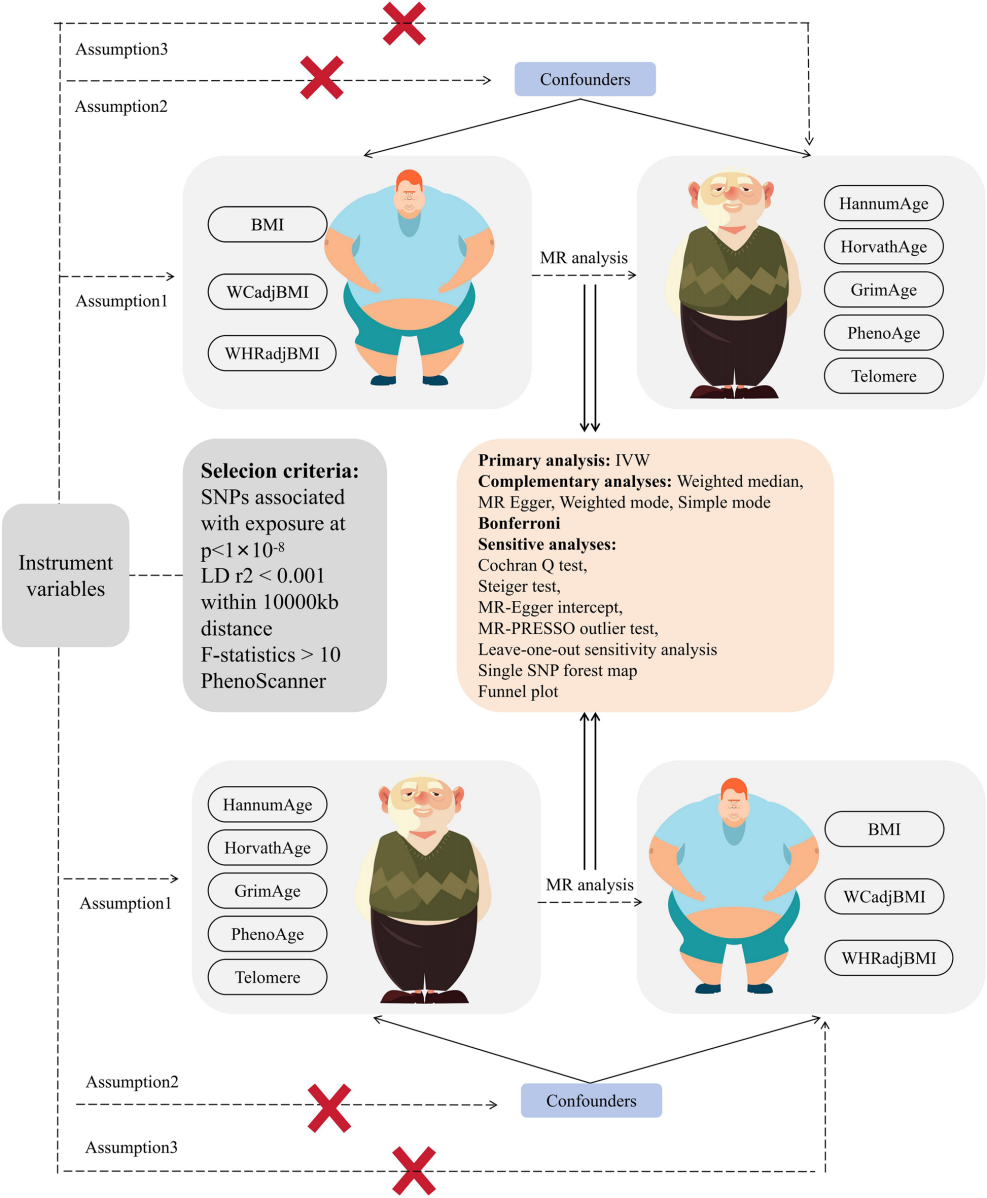
Idea map for the design of this study

MR is an epidemiologic and genetic research methodology that uses instrumental variables (IVs) based on genetic variation to explore causal associations between exposures and outcomes [31]. According to Mendel's second law, alleles are randomly assigned during gamete formation, thus the results of MR studies are not affected by confounding factors [32] and are of high quality. Based on the described situation in the previous section, a full two-way Mendelian randomization study was conducted. The aim was to thoroughly investigate the causal associations of both obesity and abdominal obesity with epigenetic age and telomere length. The aim was to resolve the previously mentioned points of contradiction in previous studies between obesity and biological age and to fill in the gaps in these studies. Such an approach provides a robust framework for understanding the intricate relationships between these variables and shedding light on their potential causal links. Flowchart of this study (Fig. 1).

## Materials and Methods

### Reporting guidelines

The present study strictly adheres to the STROBE-MR guidelines [33].

### Research design

In the present study, obesity was used as the "exposure" variable, including BMI, WC adjusted for BMI (WCadjBMI), and WHR adjusted for BMI (WHRadjBMI). Then, epigenetic age (including HannumAge, HorvathAge, GrimAge, and PhenoAge) and telomere length were adopted as “outcome” variables. The IVs were screened for MR analysis, the consistency of the study was assessed using Cochran Q, and the reliability of the results was verified by means of sensitivity analysis through horizontal multiplicity analysis and "leave-one-out" analysis. Additionally, a reverse MR analysis was performed using epigenetic age (HannumAge, HorvathAge, GrimAge, PhenoAge) and telomere length as "exposure" variables and BMI, WC, and WHR as "outcome" variables to determine the inverse relationship. MR research needs to fulfill three core assumptions: (1) IV must be highly correlated with exposure; (2) IV should be independent of any confounding factors related to exposure and outcome; and (3) IV can only affect results through exposure. In the present study, a two-way Mendelian randomization study was conducted to assess the causal association of obesity with epigenetic age and telomeres.

### Data sources

Three phenotypes of obesity were used in the present study, with IVs of BMI from a meta-analysis of 681,275 individuals of European ancestry by Genetic Investigation of Anthropometric Traits (GIANT) [34]. The case data came primarily from two consortia: UK Biobank and GIANT consortium. IVs for both WCadjBMI and WHRadjBMI were derived from a meta-analysis of 224,459 individuals of European ancestry from the GIANT Consortium. A recent meta-analysis, involving 28 cohorts and a total of 34,710 individuals of European descent, successfully identified 137 loci associated with DNA methylation biomarkers of aging. Through the study, instrumental variables regarding epigenetic age could be obtained (HannumAge, HorvathAge, GrimAge, and PhenoAge). Pooled telomere length data were obtained from the largest Genome-wide association study (GWAS) to date containing 472,174 individuals of European ancestry from UK Biobank [35]. In the aforementioned study, although there was a partial sample overlap between the GWAS of BMI and all the GWAS of telomeres, the extent of overlap was negligible. This is attributed to the fact that the GWAS sample for telomeres consisted of participants aged 45–69 years recruited from 2006–2010. In contrast, the GWAS data for BMI only included a partial sample from the UK Biobank, comprising participants aged 40–69 years and recruited from the establishment of the UK Biobank until the present day. Additionally, a meticulous screening process for weakly instrumented variables was implemented to exclude any potential overlap, confirming its negligible impact [34, 35]. Furthermore, no sample overlap occurred in other datasets. (Table 1). All of the aforementioned data have been widely used in previous MR studies [36–40] and have a high degree of reliability.

**Table 1:** Data description of obesity, epigenetic age and telomere length

Type	Phenotype	Sample source	Population	SNPs	Simple size	PubMed ID
Obesity	BMI	GIANT	Europeans	2336260	681275	30124842
	WCadjBMI	GIANT	Europeans	2566630	224459	25673412
	WHRadjBMI	GIANT	Europeans	2562516	224459	25673412
Epigenetic age	HannumAge	Centre for Cognitive Ageing and Cognitive Epidemiology	Europeans	7565045	34710	34187551
	HorvathAge		Europeans	7567532	34710	34187551
	PhenoAge		Europeans	7567585	34710	34187551
	GrimAge		Europeans	7567701	34710	34187551
Telomere	Telomere	UK Biobank	Europeans	20134421	472174	37117760

### Screening of instrumental variables

To screen qualified single-nucleotide polymorphisms (SNPs), a series of parameter settings were set. Since the SNPs for MR studies must be closely related to the exposure, the present study was screened with *p* < 5 × 10^–8^ as the threshold, we chose not to use the SNP proxy and set r2 < 0.001 and kiobase pair (kb) to 10,000, thereby preventing the effect of linkage disequilibrium (LD). Furthermore, we meticulously examined each SNP in the PhenoScanner database, specifically targeting the elimination of potential confounding factors like gender, smoking, alcohol consumption, and physical activity (*p* < 1 × 10^–5^). This thorough screening aimed to minimize bias, ensuring the robustness and stability of our results [41]. At the same time, to prevent the weak instrumental variables from biasing the outcome, the completed SNPs were screened based on F-statistic assessment. The formula is as follows [42]:

$$F=\frac{{R}^{2}\left(n-1-k\right)}{\left(1-{R}^{2}\right)k}$$

$${R}^{2}=\frac{2\times MAF \left(1-MAF\right)\times {\beta }^{2}}{{SE}^{2}\times n}$$

where MAF = minor allele frequency; β = effect size; SE = standard error; n = sample size; and k = number of instrumental variables. Screening was performed using F > 10 as a threshold [43]. The exposed SNPs for which the screening was completed were matched with the results. If a palindromic SNP was generated, it was excluded from consideration, resulting in the selection of the final instrumental variables.

### Statistical analysis

Two-way Mendelian randomization analysis was performed using the TwoSampleMR package of R software (version: 4.2.3). To determine the causal association of obesity with epigenetic age and telomeres, IVW, Weighted median, MR Egger, Weighted mode and Simple mode methods were used. IVW is the predominant method of MR analysis, which assumes that each SNP is valid and allows for a reliable assessment of the causal effect of exposure on outcomes [44]. MR Egger regression analysis is a valuable method for making causal inferences, particularly when dealing with potential pleiotropy or when a large number of instrumental variables may not be entirely valid [45]. The weighted median approach is another useful MR analysis technique, especially when you assume that at least 50% of the instrumental variables are valid [46]. Additionally, the weighted mode and simple mode methods offer more relaxed assumptions but may have lower testing efficacy compared to the previous three methods [44]. These methods can serve as supplementary tools for MR analysis, providing a broader perspective on causal relationships. A *p*-value less than 0.05 indicates statistical significance. To address the issue of multiple testing, we applied the Bonferroni correction, resulting in more stringent *p*-values. Specifically, *p*-values below 1.67 × 10^–3^ (calculated as 0.05 divided by 15, then by 2, where 2 represents two-way MR analysis) are considered compelling evidence of robust causality.

### Sensitivity analysis

To ensure the quality of the results of the present study, several heterogeneity analyses were performed using the MR-PRESSO software package: (1) The heterogeneity of SNPs was assessed using the Cochran Q test. (2) The presence of pleiotropy of SNPs was detected using the MR-Egger intercept test. (3) Mendelian randomization pleiotropy residual sum and outlier (MR-PRESSO) was used to test whether there were significant outliers in the study results, and if outliers were detected, they were removed, and the MR analysis was rerun. (4) Leave-one-out sensitivity test was conducted to observe whether there were significant changes after removing each SNP. (5) Additionally, we employed the MR-Steiger directionality test to evaluate the potential causal relationship between the assumed exposure and anticipated outcomes.

## Results

### Instrumental variables

In the present study, sufficient obesity-wide loci were screened for MR analysis (BMI IVs = 490, interval of F-statistic = 28.62—1426.17; WCadjBMI IVs = 45, interval of F-statistic = 29.75—448.44; WHRadjBMI IVs = 31, interval of F-statistic = 29.22—169.79) (Supplementary Table 1). Additionally, sufficient epigenetic age and telomere-wide loci were screened for reverse MR analysis (HannumAge IVs = 9, interval of F-statistic = 30.82—98.90; HorvathAge IVs = 24, interval of F-statistic = 30.82—98.90; GrimAge IVs = 4, interval of F-statistic = 31.08—239.74; PhenoAge IVs = 11, interval of F-statistic = 30.77—89.39; Telomere IVs = 4, interval of F-statistic = 29.86—1628.82) (Supplementary Table 2). The F-statistic for each SNP was > 10, suggesting a low likelihood of the presence of weak instrumental variables, and that these IVs could make good inferences about the causal relationship between exposure and outcome variables. In this bidirectional MR analysis, it was observed that a small number of palindromic SNPs and outlier SNPs were present (as indicated in Supplementary Table 3). To maintain the integrity and reliability of the final MR analysis, these outliers were excluded from the analysis.

### Causal effects of obesity on epigenetic age

**Fig. 2:**
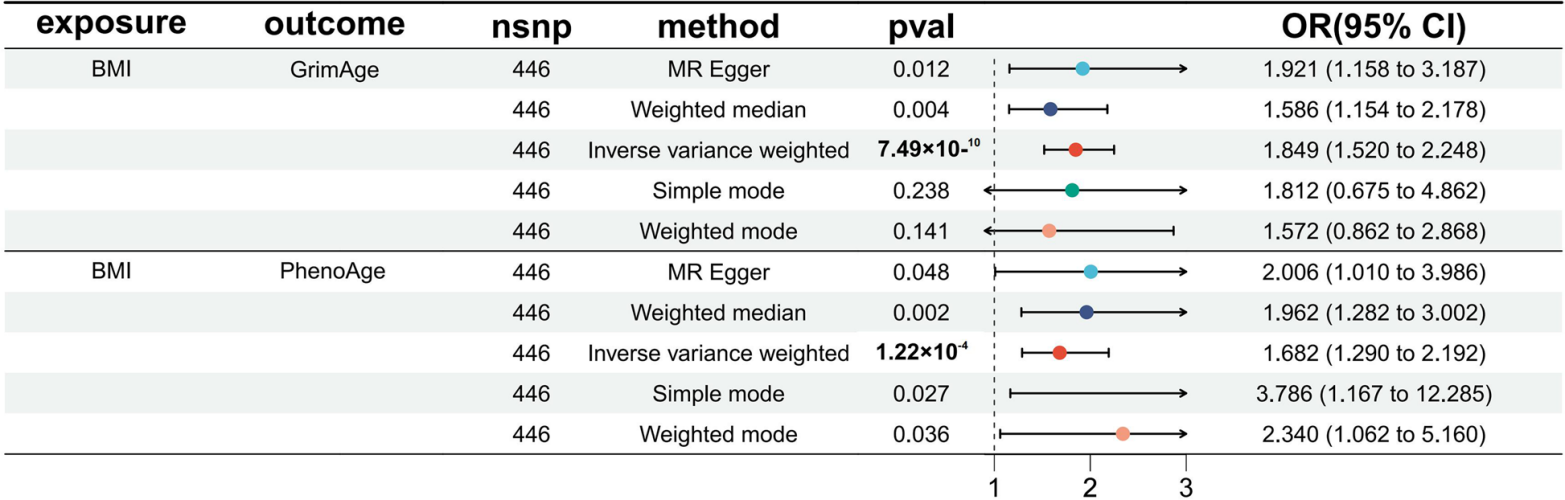
MR estimations of the impact of BMI on GrimAge and PhenoAge

Using MR analysis, a partial causal relationship between obesity and acceleration of epigenetic age was observed. IVW modeling shows a significant causal relationship between BMI and acceleration of GrimAge (OR: 1.849, 95% confidence interval (CI): 1.520—2.248, *p* = 7.49 × 10^–10^) and PhenoAge (OR: 1.682, 95%CI: 1.290—2.192, *p* = 1.22 × 10^–4^). WCadjBMI had a significant causal relationship with the acceleration of HannumAge (OR:1.554, 95%CI: 1.092—2.211, *p* = 0.014), HannumAge (OR:1.554, 95%CI: 1.092—2.211, *p* = 0.014) and PhenoAge (OR: 2.099, 95%CI: 1.248—3.531, *p* = 0.005). WHRadjBMI had a significant causal relationship with PhenoAge (OR: 1.974, 95%CI: 1.053—3.702, *p* = 0.034) acceleration. The ORs and 95% CIs obtained from the MR analyses between the three obesity exposures included in the present study and other epigenetic clocks were not statistically significant (Supplementary Table 4). However, following Bonferroni correction for multiple testing, only the MR analysis results for BMI with GrimAge and PhenoAge remained statistically significant (*p* < 1.67 × 10^–3^), indicating robust causal associations (Fig. 2). The study employed scatter plots (Supplementary Figs. 1 and 2) to illustrate the causal relationship between obesity and epigenetic age. To provide a comprehensive visualization of the findings, we included leave-one-out sensitivity analysis plots, forest plots for MR analysis of individual SNPs, and funnel plots (Supplementary Figs. 3 and 8). Moreover, the Steiger test for directionality resulted in TRUE (*p* < 0.05) in our study. The Cochran Q test revealed minimal heterogeneity among SNPs when comparing the IVW and MR-Egger methods (Supplementary Table 5). Additionally, the MR-Egger intercept indicated the absence of horizontal pleiotropy (Supplementary Table 5).

### Causal effects of obesity on telomeres

**Fig. 3:**
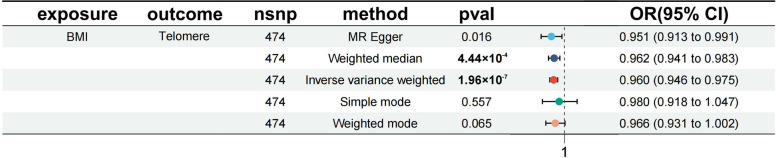
MR estimations of the impact of Obesity on Telomere

Using MR analysis, a partial causal relationship between obesity and telomere length shortening was observed. IVW modeling shows a significant causal relationship between BMI and telomere length shortening (OR: 0.960, 95%CI: 0.946—0.975, *p* < 0.001). WCadjBMI was also significantly and causally associated with telomere length shortening (OR: 0.965, 95%CI: 0.936—0.995, *p* = 0.021). The causal relationship between WHRadjBMI and the shortening of telomere length (OR: 0.978, 95%CI: 0.930—1.029, *p* = 0.397) was not statistically significant but the direction still showed a negative correlation (Supplementary Table 4). However, following Bonferroni correction for multiple testing, only the MR analysis results for BMI compared to telomere length remained statistically significant (*p* < 1.67 × 10^–3^), indicating a robust causal association (Fig. 3). Scatter plots were used to visualize the causal relationship between obesity and telomere length (Supplementary Fig. 9). For a comprehensive visualization of the results, the leave-one-out sensitivity analysis plots were supplemented, as well as the forest plots for MR analysis of individual SNPs and the funnel plots (Supplementary Figs. 10 and 12). Moreover, the Steiger test for directionality resulted in TRUE (*p* < 0.05) in our study. The Cochran Q test results show some heterogeneity between the SNPs from the IVW method and the MR-Egger method (Supplementary Table 6). However, the MR-Egger intercept shows no horizontal pleiotropy (Supplementary Table 6).

### Causal relationship between epigenetic age and obesity (reverse MR analysis)

**Fig. 4:**
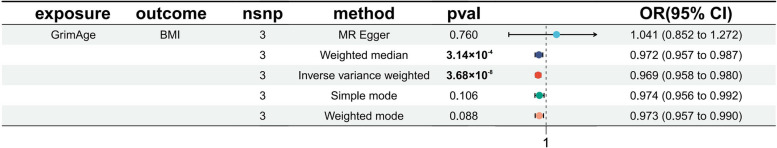
MR estimations of the impact of GrimAge on Obesity

Using inverse MR analysis, only a slight causal relationship between epigenetic age and obesity was observed. The IVW model shows a statistically significant difference in the causal relationship between HorvathAge and BMI (OR: 1.012, 95%CI: 1.004—1.019, *p* = 0.002). The causal relationship between GrimAge and BMI (OR: 0.969, 95%CI: 0.958—0.980, *p* < 0.001) was statistically significant, as was that between PhenoAge and BMI (OR: 1.007, 95%CI: 1.001—1.014, *p* = 0.027). The results of the reverse MR analysis between the other epigenetic clocks and the three obesity indicators included in the present study were not statistically significant (Supplementary Table 4). However, following Bonferroni correction for multiple testing, only the MR analyses of GrimAge compared to BMI exhibited statistical significance (*p* < 1.67 × 10^–3^) after the correction, indicating a robust causal association (Fig. 4). The aim was to establish a causal relationship between epigenetic age and obesity through the utilization of scatter plots for visualization (Supplementary Fig. 13). To provide a comprehensive representation of the findings, additional visual aids were included, such as leave-one-out sensitivity analysis plots, forest plots for MR) analysis of individual SNPs and funnel plots (Supplementary Figs. 14 and 16). Moreover, the Steiger test for directionality resulted in TRUE (*p* < 0.05) in our study. The results of the Cochran Q test indicate the absence of significant heterogeneity between SNPs derived from both the IVW and MR-Egger methods (Supplementary Table 7). Further, the MR-Egger intercept demonstrated the absence of horizontal pleiotropy (Supplementary Table 7).

### Causal relationship between telomeres and obesity (reverse MR analysis)

Through inverse MR analysis, an observation was made that the causal relationship between telomeres and obesity was not statistically significant (Supplementary Table 4). None of the individual results meet the criteria for statistical significance after applying Bonferroni's multiple test correction. Moreover, the Steiger test for directionality resulted in TRUE (*p* < 0.05) in our study. The results of the Cochran Q test reveal the absence of significant heterogeneity between SNPs derived from both the IVW and MR-Egger methods (Supplementary Table 8). Additionally, the MR-Egger intercept demonstrated the absence of horizontal pleiotropy (Supplementary Table 8).

## Discussion

Aging is an unavoidable biological process that is accompanied by a decline in organ function and an increase in environmental susceptibility. As such, research on anti-aging mechanisms has been a key area of interest in the field of medicine [47]. Telomere length and epigenetic age can be used as indicators of aging. A telomere is a piece of DNA and protein complex located at the end of a chromosome that functions to protect DNA from damage after replication [48]. The protective caps at the end of chromosomes, naturally shorten with each cell division throughout an individual's life. This process is a hallmark of cellular aging and is linked to various health conditions, including cancer. Telomere length serves as a valuable indicator of an individual's aging process and overall health [49, 50]. Epigenetic age is a class of biological age metrics distinct from telomeres, which are derived based on DNAm and are extensively employed to measure the level of cellular and tissue senescence in organisms [18]. The acceleration of epigenetic age is frequently employed to characterize individuals whose epigenetic age surpasses their chronological age, indicating a diminished state of health in the individual [51]. The total number of obese patients is increasing globally every year [1]. The disorders of lipid metabolism induced by obesity can contribute to cognitive impairment in humans through various lipid metabolic pathways, including cholesterol, triglycerides, apolipoprotein, lipoprotein(a), and low-density lipoprotein cholesterol [52]. Exploring the causal connection between obesity and the acceleration of epigenetic aging, along with the reduction in telomere length, represents a pioneering and insightful research avenue that may yield strategies for mitigating the aging process in humans.

To the present knowledge, the present study is the first comprehensive bi-directional causal study of obesity with epigenetic age and telomeres. Our study addresses the contradictions observed in previous correlation studies between obesity and biological age and effectively addresses the shortcomings of prior Mendelian randomization studies in this domain. Findings were made that BMI had a significant positive causal relationship with acceleration of GrimAge and PhenoAge, but not with acceleration of HannumAge and HorvathAge. WCadjBMI had a significant positive causal relationship with acceleration of HannumAge, GrimAge and PhenoAge. WHRadjBMI exhibited a significant association solely with the acceleration of PhenoAge. Overall, all three obesity indicators included in the study were significantly associated with the acceleration of PhenoAge; however, none were associated with the acceleration of HorvathAge. Moreover, the causal association between WCadjBMI and PhenoAge acceleration was the strongest among these causal associations (OR: 2.099, 95%CI: 1.248—3.531, *p* = 0.005). Such findings could be attributed to the fact that the PhenoAge clock is projected to include C-reactive protein, Glucose, White blood cell count and other indicators that are susceptible to lifestyle behaviors, which have been shown to be strongly associated with obesity and body composition [53–55]. The estimation of the GrimAge clock incorporates the measurement of plasma leptin [21], which is mostly produced in white adipose tissue, and its secretion is significantly and positively correlated with adipose tissue and adipocyte volume [56]. Estimates from the HannumAge clock incorporate the proportions of cytotoxic T cells, helper T, natural killer, B cells, and granulocytes in whole blood samples, and the state of obesity can have an impact on the number and activation of these immune cells [57]. The three obesity indicators examined did not demonstrate a statistically significant acceleration effect on HorvathAge. This aligns with findings from a cross-sectional study conducted in Taiwan with a sample size of 2474, which highlighted the lack of significant correlation between obesity and HorvathAge [9]. The weaker predictive performance of HorvathAge in terms of lipids, glucose, and other blood sample metrics compared to other epigenetic age tools [22] might be attributed to its inadequate training on relevant blood metrics. However, a positive trend was observed in the relationship. Regarding the association between obesity and telomere length, all three indicators considered in the study displayed a negative correlation with telomere length, but only BMI and WCadjBMI exhibited a statistically significant association with shorter telomere length. Such results are generally consistent with previous observational studies and meta-analyses [58–60]. This may be due to oxidative stress in adipose tissue of obese individuals, and excessive oxidative stress potentially leading to accelerated shortening of telomeres [43]. Moreover, obesity is frequently characterized as a systemic, chronic, low-grade inflammatory condition, leading to an upregulation in the expression of pro-inflammatory cytokines like interleukin-1 and tumor necrosis factor-α in the adipose tissue of obese individuals [61]. This phenomenon may be attributed, in part, to the hyperpolarization of M1-type macrophages, among other factors [62, 63]. Such accumulation of inflammation promotes telomere shortening [60]. Additionally, the causal associations between BMI and GrimAge, PhenoAge, and telomere length were consistent with *p*-values corrected by the Bonferroni multiple test, demonstrating increased robustness compared to other results. This robustness may stem from GrimAge and PhenoAge's stronger correlation with clinical lipid profiles, blood pressure, and glucose indices compared to the primary representative epigenetic age tool [22]. This underscores the significance of BMI as an obesity measure, potentially attributed to its broader coverage of various obesity types compared to WCadjBMI and WHRadjBMI. BMI stands out as a straightforward and practical tool for obesity assessment. However, it is acknowledged that BMI might be somewhat biased as it fails to differentiate between central and peripheral obesity, with the latter demonstrating protective effects against hypertension, type 2 diabetes mellitus, dyslipidemia, and cardiovascular diseases through subcutaneous fat accumulation in the legs and arms [64–67].

In the reverse MR analysis, only HorvathAge, GrimAge and PhenoAge were found to have significant causal relationships with BMI, but they were in different directions. Acceleration of HorvathAge and PhenoAge was causally associated with decreased BMI, and acceleration of GrimAge was causally associated with increased BMI. Although there was statistical significance in the results, the effects of these 3 age clocks on BMI were small, and the relationships were trivial compared with their positive causal associations. Such findings could likely be attributed to confounding factors that complicate the associations between these three epigenetic clocks and BMI, even when rigorous screening conditions are applied, such as economic status. Additionally, the observed outcomes may be influenced by limitations in statistical power. In MR analyses assessing the causal link between telomeres and obesity, it was determined that telomere shortening does not induce changes in the obesity phenotype.

In the present study, the bidirectional causal associations of obesity with epigenetic age and telomeres were comprehensively analyzed for the first time, and the use of MR analysis was beneficial in mitigating the effects of confounding factors. In addition, an adequate sensitivity analysis was conducted to support the quality of the findings. The present study validates the findings from prior observational research and offers a robust evaluation of contradictory results from previous cross-sectional investigations. Furthermore, this study serves as a valuable complement to MR analyses conducted in the same field. These strengths provide support for the robustness and reliability of the present results and suggest that a causal relationship was established between obesity, epigenetic age and telomeres.

There present study had several unavoidable limitations. Firstly, the data for the present study came from a European population. While achieving individual homogeneity can effectively mitigate bias arising from group differences and yield dependable results for MR analysis, it raises controversy regarding the generalizability of our findings to ethnic groups beyond Europe. Hence, future studies should focus on targeted homogeneity within other human races to validate the applicability of our study's results. The study focused exclusively on diagnostic indicators of obesity and abdominal obesity, without considering more detailed measures of body composition, such as the percentage of body fat. As previously mentioned, various fat deposits in distinct regions may exert diverse impacts on health and aging. Futhermore, a more comprehensive investigation in the future could incorporate these additional aspects. As the field of epigenetics continues to advance, with the development of new aging markers and improvements in relevant GWAS data, there is potential to broaden the scope of research to explore bidirectional causal relationships between these markers and obesity.

## Conclusion

In conclusion, the present study supports a strong causal association between obesity, accelerated epigenetic aging and telomere length shrinkage. The causal relationship between WCadjBMI and PhenoAge acceleration was the strongest among them. However, none of the three obesity indicators (BMI, WCadjBMI and WHRadjBMI) included in the study had a significant causal relationship with acceleration of HorvathAge. In the reverse MR analysis, a statistically significant causal association was identified between HorvathAge, PhenoAge, GrimAge, and BMI, but the strength of this causal association, as indicated by their ORs, was relatively low. Nevertheless, the present study unveils the causal link between obesity and biological age markers, offering valuable insights for scientific research aimed at delaying the aging process in humans.

### Supplementary Information

**Additional file 1.  **Code for this study

**Additional file 2:Supplementary Table 1.** Obesity instrumental variables, **Supplementary Table 2.** Epigenetic age and Telomere instrumental variables, **Supplementary Table 3.** Palindromic SNPs and Outlier SNPs, **Supplementary Table 4.** Results of MR analysis of causal association of obesity with epigenetic age and telomeres, **Supplementary Table 5.** Heterogeneity and pleiotropy of Obesity and Epigenetic age, **Supplementary Table 6.** Heterogeneity and pleiotropy of Obesity and Telomere, **Supplementary Table 7.** Heterogeneity and pleiotropy of Epigenetic age and Obesity, **Supplementary Table 8.** Heterogeneity and pleiotropy of Telomere age and Obesity

**Additional file 3:Supplementary Figure 1.** Scatterplot of MR analysis of BMI on GrimAge, **Supplementary Figure 2.** Scatterplot of MR analysis of BMI on PhenoAge, **Supplementary Figure 3.** The leave-one-out analysis for BMI on GrimAge, **Supplementary Figure 4.** The leave-one-out analysis for BMI on PhenoAge, **Supplementary Figure 5.** The single SNP analysis for BMI on GrimAge, **Supplementary Figure 6.** The single SNP analysis for BMI on PhenoAge, **Supplementary Figure 7.** The funnel plots for BMI on GrimAge, **Supplementary Figure 8.** The funnel plots for BMI on PhenoAge, **Supplementary Figure 9.** Scatterplot of MR analysis of BMI on Telomere, **Supplementary Figure 10.** The leave-one-out analysis for BMI on Telomere**, Supplementary Figure 11.** The single SNP analysis for BMI on Telomere, **Supplementary Figure 12.** The funnel plots for BMI on Telomere, **Supplementary Figure 13.** Scatterplot of MR analysis of BMI on Telomere, **Supplementary Figure 14.** The leave-one-out analysis for GrimAge on BMI, **Supplementary Figure 15.** The single SNP analysis for GrimAge on BMI, **Supplementary Figure 16.** The funnel plots for GrimAge on BMI

## Data Availability

Publicly available datasets were analyzed in this study. This data can be found here: All GWAS data used in this study are available in the IEU open GWAS project (https://gwas.mrcieu.ac.uk/) and Edinburgh DataShare (https://datashare.is.ed.ac.uk/handle/10283/3645).
