# Supplementary material for: Causal association of obesity with epigenetic aging and telomere length: a bidirectional mendelian randomization study
Source: Lipids Health Dis. 2024 Mar 12;23:78. doi: 10.1186/s12944-024-02042-y (PMC10935937; doi:10.1186/s12944-024-02042-y)
Supplement: Supplementary file 1 — Additional file 1. Code for this study [file 12944_2024_2042_MOESM1_ESM.docx]

**Code for this study**

**#The code provided illustrates the process using exposure, such as BMI, and outcome, represented by GrimAge. This example serves to demonstrate the code application, and the same code can be employed for the other Mendelian randomization analyses conducted in this paper.**

install.packages("devtools")

devtools::install_github("MRCIEU/TwoSampleMR")

library(TwoSampleMR)

install.packages("data.table")

library(data.table)

getwd()

BMI_exp<- extract_instruments(outcomes = 'ieu-b-40')

extract_instruments(outcomes,p1 = 5e-08,clump = TRUE,

p2 = 5e-08,r2 = 0.001,kb = 10000,access_token = ieugwasr::check_access_token(),

force_server = FALSE)

dim(BMI_exp)

write.csv(BMI_exp,"BMI_exp.csv")

library(data.table)

GrimAge_out <-fread('GrimAge.txt',header=T)

colnames(GrimAge_out))

head(GrimAge_out)

GrimAge_out$phenotype <- 'GrimAge'

#HF_out$beta <- log(out$OR)

#HF_out$se <-abs(out$beta/qnorm(out$P_VALUE/2,lower.tail=F))

head(GrimAge_out)

GrimAge_out_dat <- format_data(

dat=GrimAge_out,

type = "outcome",

snps = GrimAge_out$MarkerName,

header = TRUE,

phenotype_col = "phenotype",

snp_col = "rsID",

beta_col = "Effect",

se_col = "SE",

effect_allele_col = "A1",

other_allele_col = "A2",

pval_col = "P",

ncase_col = "N",

#ncontrol_col = "N_CONTROLS",

chr_col = "chr",

pos_col = "pos")

head(GrimAge_out_dat)

write.csv(GrimAge_out_dat,"GrimAge_out_dat.csv")

BMI_GrimAge_mydata <- harmonise_data(exposure_dat=BMI_exp,

outcome_dat=GrimAge_out_dat, action= 3)

write.csv(BMI_GrimAge_mydata,"BMI_GrimAge_mydata.csv")

devtools::install_github("rondolab/MR-PRESSO",force = TRUE)

library(MRPRESSO)

colnames(BMI_GrimAge_mydata)

mr_presso(BetaOutcome ="beta.outcome",

BetaExposure = "beta.exposure",

SdOutcome ="se.outcome",

SdExposure = "se.exposure",

OUTLIERtest = TRUE,

DISTORTIONtest = TRUE,

data = BMI_GrimAge_mydata,

NbDistribution = 1000,

SignifThreshold = 0.05)

BMI_GrimAge_mydata_t <- BMI_GrimAge_mydata[-c(), ]

BMI_GrimAge_res <- mr(BMI_GrimAge_mydata_t)

BMI_GrimAge_res

write.csv(BMI_GrimAge_res,"res_BMI_GrimAge.csv",row.names = F)

BMI_GrimAge_res_single <- mr_singlesnp(BMI_GrimAge_mydata_t)

BMI_GrimAge_res_single_ORR <-generate_odds_ratios(BMI_GrimAge_res_single)

write.csv(BMI_GrimAge_res_single_ORR,"ORR_single_BMI_GrimAge.csv")

BMI_GrimAge_het <- mr_heterogeneity(BMI_GrimAge_mydata_t)

BMI_GrimAge_het

write.csv(BMI_GrimAge_het,"het_BMI_GrimAge.csv")

BMI_GrimAge_pleio <- mr_pleiotropy_test(BMI_GrimAge_mydata_t)

BMI_GrimAge_pleio

write.csv(BMI_GrimAge_pleio,"pleio_BMI_GrimAge.csv")

BMI_GrimAge_single <- mr_leaveoneout(BMI_GrimAge_mydata_t)

mr_leaveoneout_plot(BMI_GrimAge_single)

mr_scatter_plot(BMI_GrimAge_res,BMI_GrimAge_mydata_t)

BMI_GrimAge_res_single <- mr_singlesnp(BMI_GrimAge_mydata_t)

mr_forest_plot(BMI_GrimAge_res_single)

mr_funnel_plot(BMI_GrimAge_res_single)
