# Supplementary figures and images for: Causal association of obesity with epigenetic aging and telomere length: a bidirectional mendelian randomization study
Source: Lipids Health Dis. 2024 Mar 12;23:78. doi: 10.1186/s12944-024-02042-y (PMC10935937; doi:10.1186/s12944-024-02042-y)

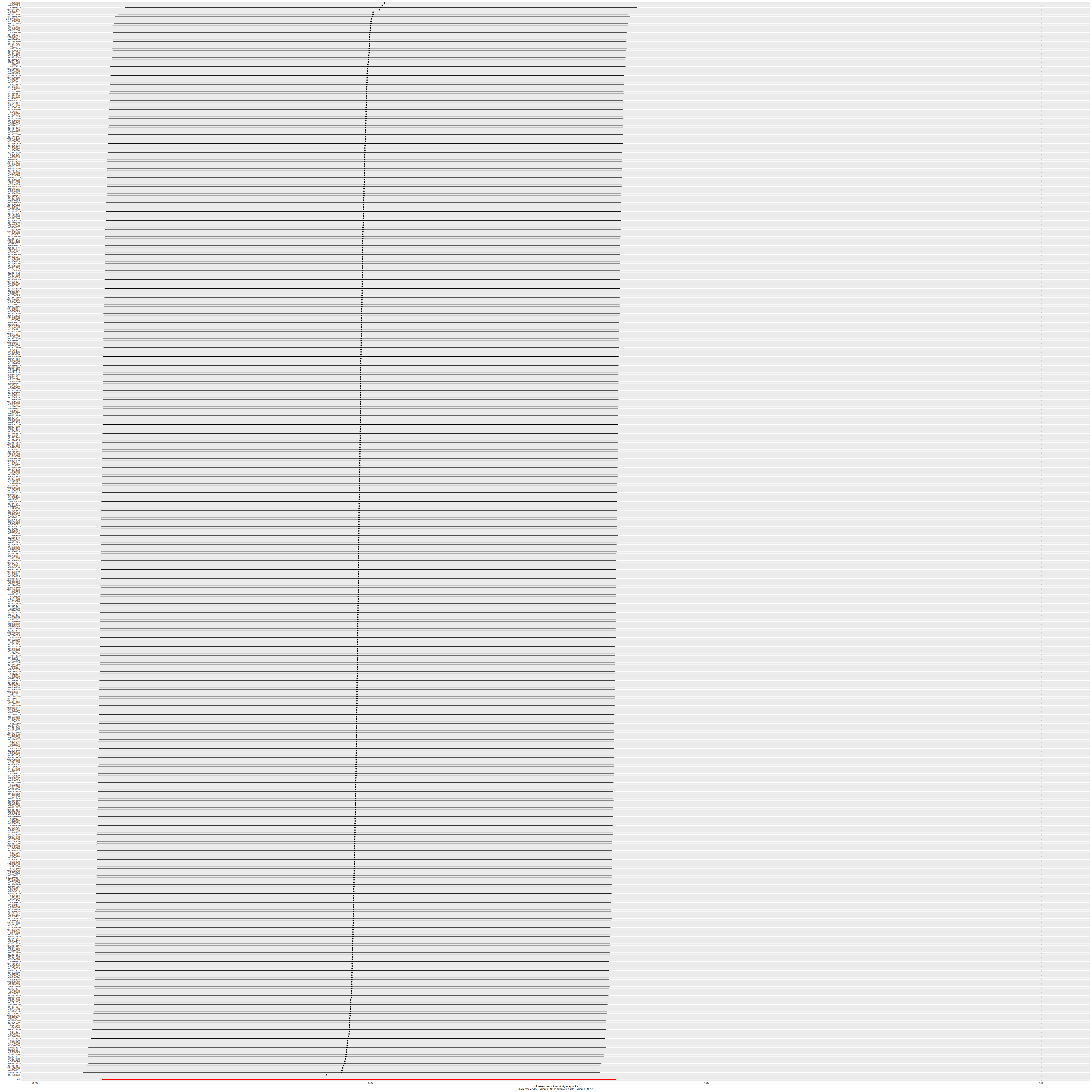

Supplement: Supplementary file 3 — Additional file 3:Supplementary Figure 1. Scatterplot of MR analysis of BMI on GrimAge, Supplementary Figure 2. Scatterplot of MR analysis of BMI on PhenoAge, Supplementary Figure 3. The leave-one-out analysis for BMI on GrimAge, Supplementary Figure 4. The leave-one-out analysis for BMI on PhenoAge, Supplementary Figure 5. The single SNP analysis for BMI on GrimAge, Supplementary Figure 6. The single SNP analysis for BMI on PhenoAge, Supplementary Figure 7. The funnel plots for BMI on GrimAge, Supplementary Figure 8. The funnel plots for BMI on PhenoAge, Supplementary Figure 9. Scatterplot of MR analysis of BMI on Telomere, Supplementary Figure 10. The leave-one-out analysis for BMI on Telomere, Supplementary Figure 11. The single SNP analysis for BMI on Telomere, Supplementary Figure 12. The funnel plots for BMI on Telomere, Supplementary Figure 13. Scatterplot of MR analysis of BMI on Telomere, Supplementary Figure 14. The leave-one-out analysis for GrimAge on BMI, Supplementary Figure 15. The single SNP analysis for GrimAge on BMI, Supplementary Figure 16. The funnel plots for GrimAge on BMI [file 12944_2024_2042_MOESM3_ESM.zip › Supplementary figures 1-16/Supplementary Figure 10. The leave-one-out analysis for BMI on Telomere.tif]

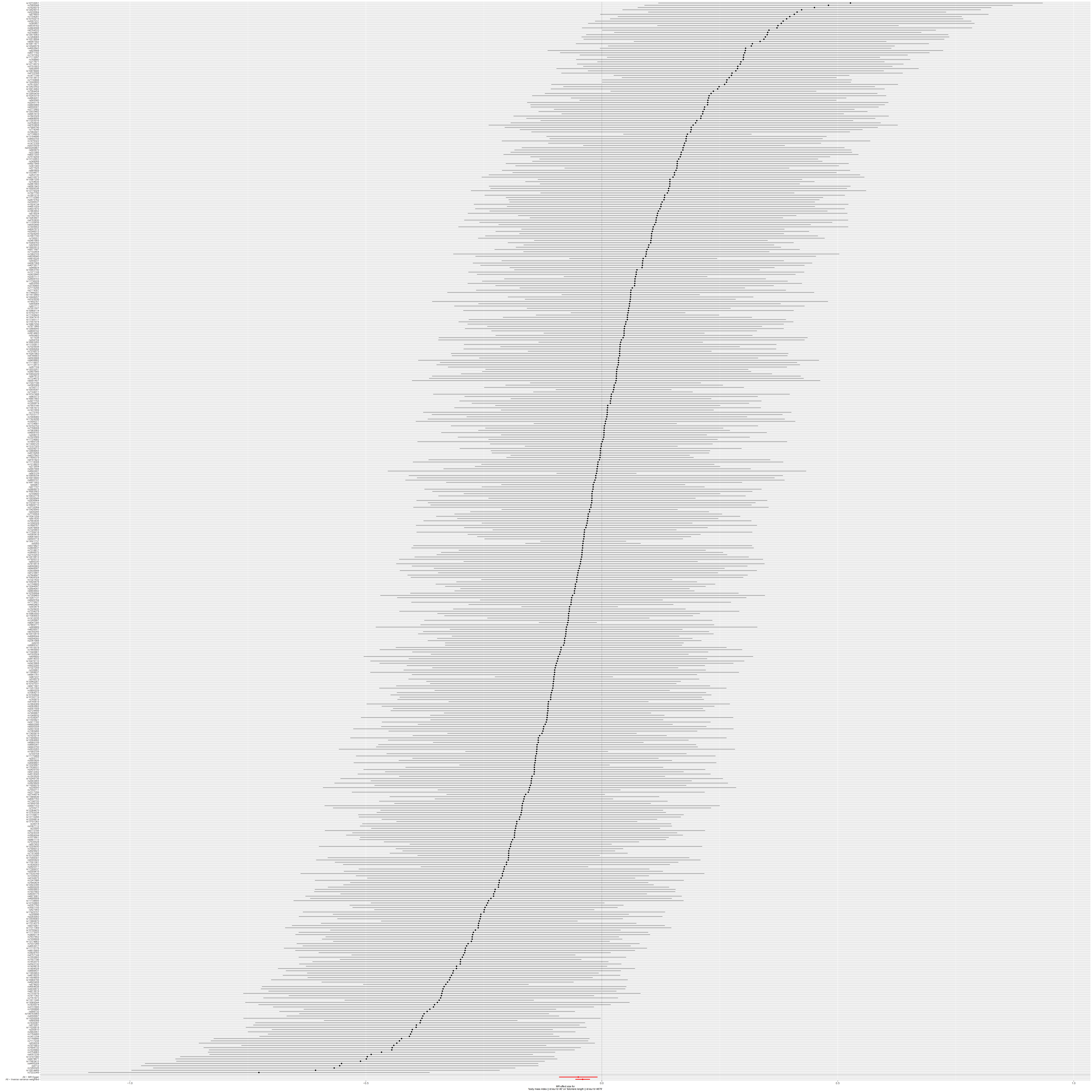

Supplement: Supplementary file 3 — Additional file 3:Supplementary Figure 1. Scatterplot of MR analysis of BMI on GrimAge, Supplementary Figure 2. Scatterplot of MR analysis of BMI on PhenoAge, Supplementary Figure 3. The leave-one-out analysis for BMI on GrimAge, Supplementary Figure 4. The leave-one-out analysis for BMI on PhenoAge, Supplementary Figure 5. The single SNP analysis for BMI on GrimAge, Supplementary Figure 6. The single SNP analysis for BMI on PhenoAge, Supplementary Figure 7. The funnel plots for BMI on GrimAge, Supplementary Figure 8. The funnel plots for BMI on PhenoAge, Supplementary Figure 9. Scatterplot of MR analysis of BMI on Telomere, Supplementary Figure 10. The leave-one-out analysis for BMI on Telomere, Supplementary Figure 11. The single SNP analysis for BMI on Telomere, Supplementary Figure 12. The funnel plots for BMI on Telomere, Supplementary Figure 13. Scatterplot of MR analysis of BMI on Telomere, Supplementary Figure 14. The leave-one-out analysis for GrimAge on BMI, Supplementary Figure 15. The single SNP analysis for GrimAge on BMI, Supplementary Figure 16. The funnel plots for GrimAge on BMI [file 12944_2024_2042_MOESM3_ESM.zip › Supplementary figures 1-16/Supplementary Figure 11. The single SNP analysis for BMI on Telomere.tif]

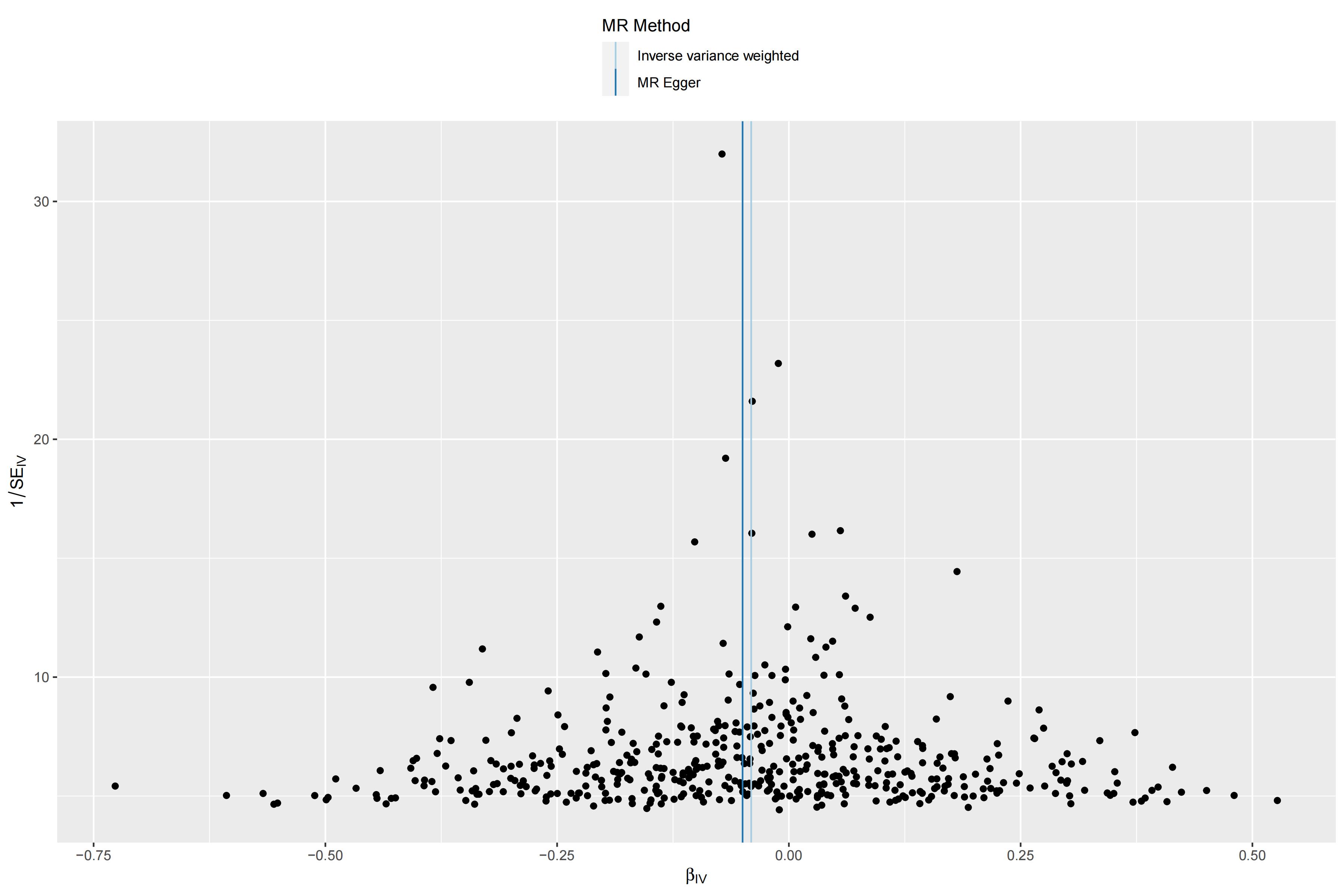

Supplement: Supplementary file 3 — Additional file 3:Supplementary Figure 1. Scatterplot of MR analysis of BMI on GrimAge, Supplementary Figure 2. Scatterplot of MR analysis of BMI on PhenoAge, Supplementary Figure 3. The leave-one-out analysis for BMI on GrimAge, Supplementary Figure 4. The leave-one-out analysis for BMI on PhenoAge, Supplementary Figure 5. The single SNP analysis for BMI on GrimAge, Supplementary Figure 6. The single SNP analysis for BMI on PhenoAge, Supplementary Figure 7. The funnel plots for BMI on GrimAge, Supplementary Figure 8. The funnel plots for BMI on PhenoAge, Supplementary Figure 9. Scatterplot of MR analysis of BMI on Telomere, Supplementary Figure 10. The leave-one-out analysis for BMI on Telomere, Supplementary Figure 11. The single SNP analysis for BMI on Telomere, Supplementary Figure 12. The funnel plots for BMI on Telomere, Supplementary Figure 13. Scatterplot of MR analysis of BMI on Telomere, Supplementary Figure 14. The leave-one-out analysis for GrimAge on BMI, Supplementary Figure 15. The single SNP analysis for GrimAge on BMI, Supplementary Figure 16. The funnel plots for GrimAge on BMI [file 12944_2024_2042_MOESM3_ESM.zip › Supplementary figures 1-16/Supplementary Figure 12. The funnel plots for BMI on Telomere.tif]

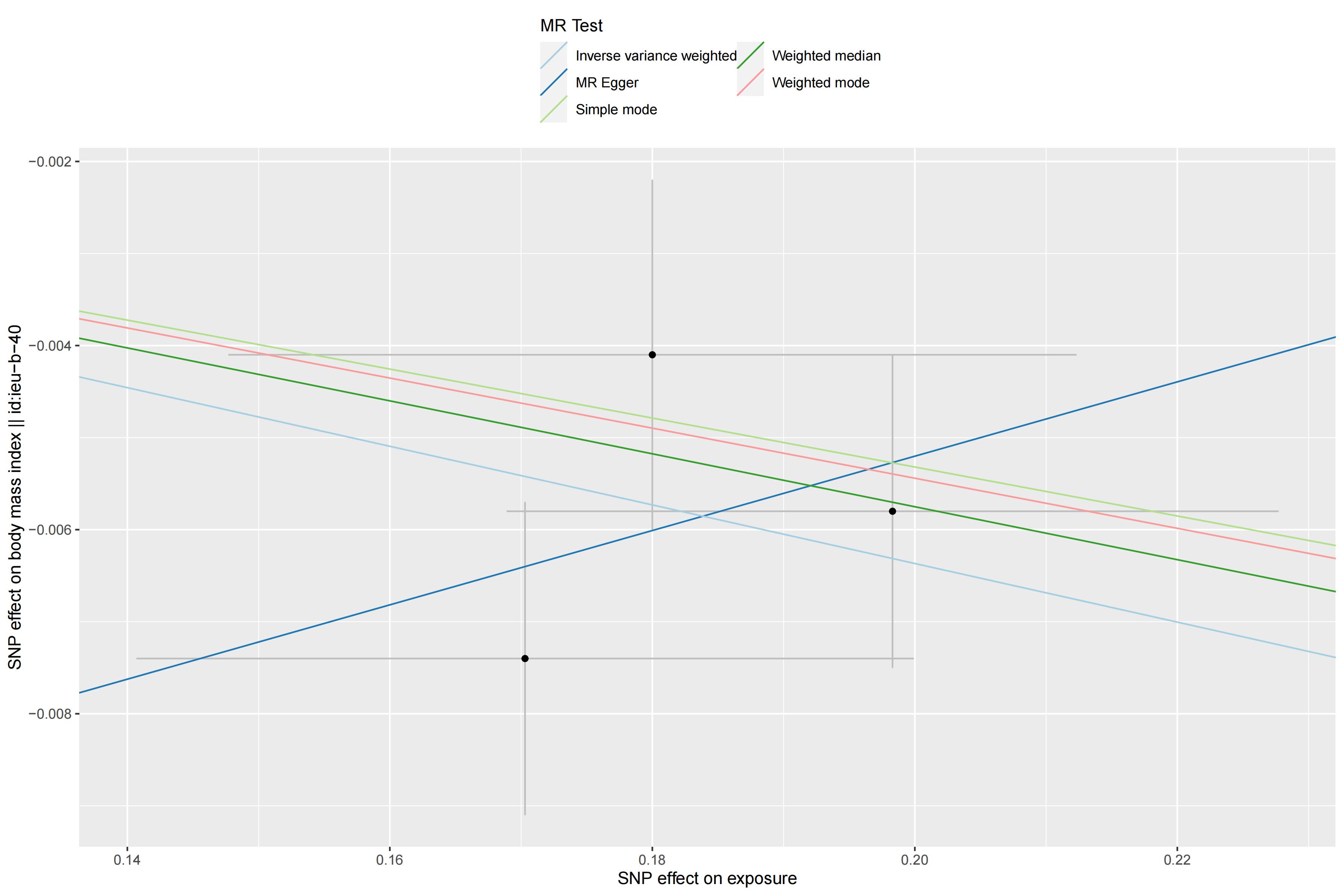

Supplement: Supplementary file 3 — Additional file 3:Supplementary Figure 1. Scatterplot of MR analysis of BMI on GrimAge, Supplementary Figure 2. Scatterplot of MR analysis of BMI on PhenoAge, Supplementary Figure 3. The leave-one-out analysis for BMI on GrimAge, Supplementary Figure 4. The leave-one-out analysis for BMI on PhenoAge, Supplementary Figure 5. The single SNP analysis for BMI on GrimAge, Supplementary Figure 6. The single SNP analysis for BMI on PhenoAge, Supplementary Figure 7. The funnel plots for BMI on GrimAge, Supplementary Figure 8. The funnel plots for BMI on PhenoAge, Supplementary Figure 9. Scatterplot of MR analysis of BMI on Telomere, Supplementary Figure 10. The leave-one-out analysis for BMI on Telomere, Supplementary Figure 11. The single SNP analysis for BMI on Telomere, Supplementary Figure 12. The funnel plots for BMI on Telomere, Supplementary Figure 13. Scatterplot of MR analysis of BMI on Telomere, Supplementary Figure 14. The leave-one-out analysis for GrimAge on BMI, Supplementary Figure 15. The single SNP analysis for GrimAge on BMI, Supplementary Figure 16. The funnel plots for GrimAge on BMI [file 12944_2024_2042_MOESM3_ESM.zip › Supplementary figures 1-16/Supplementary Figure 13. Scatterplot of MR analysis of BMI on Telomere.tif]

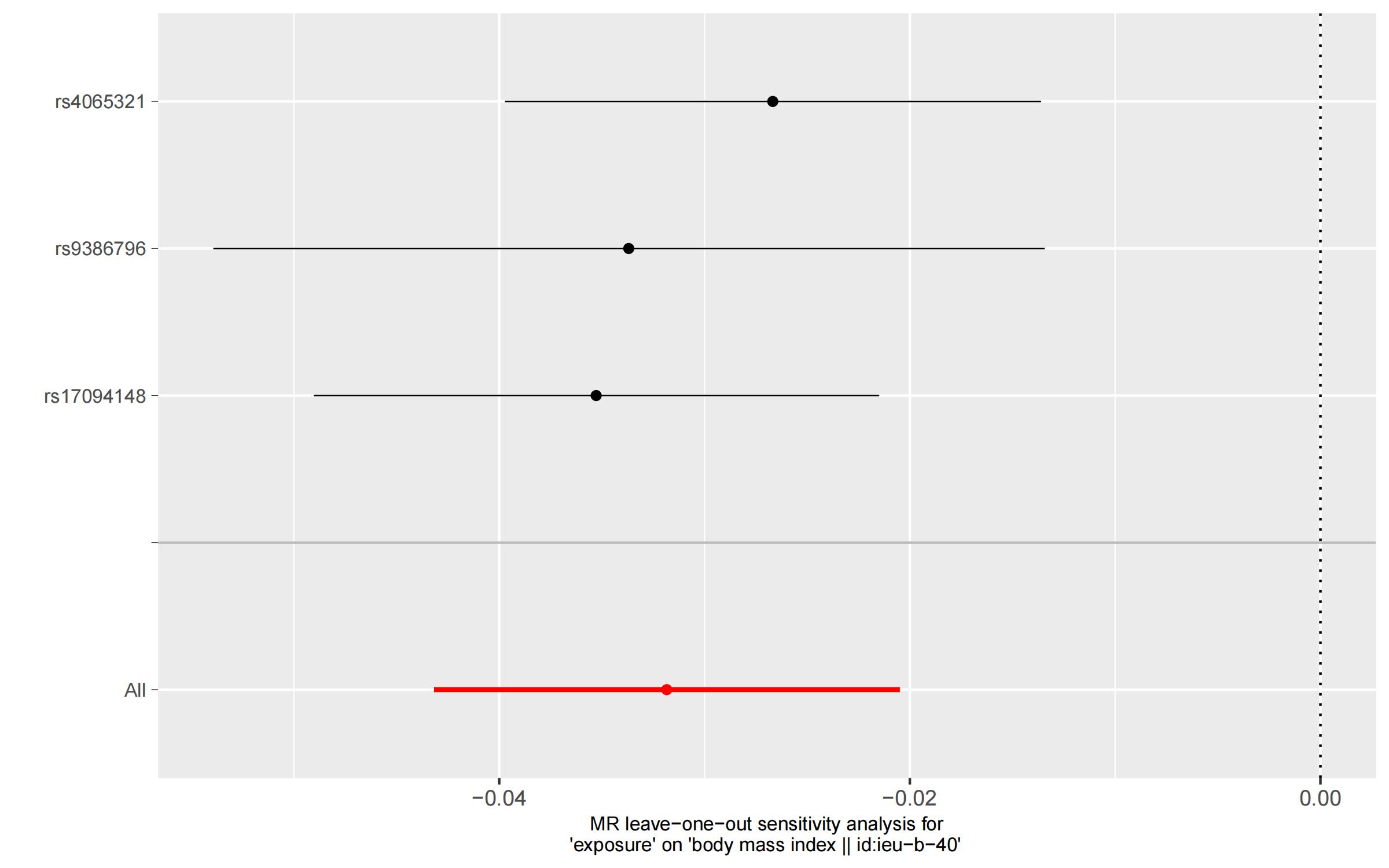

Supplement: Supplementary file 3 — Additional file 3:Supplementary Figure 1. Scatterplot of MR analysis of BMI on GrimAge, Supplementary Figure 2. Scatterplot of MR analysis of BMI on PhenoAge, Supplementary Figure 3. The leave-one-out analysis for BMI on GrimAge, Supplementary Figure 4. The leave-one-out analysis for BMI on PhenoAge, Supplementary Figure 5. The single SNP analysis for BMI on GrimAge, Supplementary Figure 6. The single SNP analysis for BMI on PhenoAge, Supplementary Figure 7. The funnel plots for BMI on GrimAge, Supplementary Figure 8. The funnel plots for BMI on PhenoAge, Supplementary Figure 9. Scatterplot of MR analysis of BMI on Telomere, Supplementary Figure 10. The leave-one-out analysis for BMI on Telomere, Supplementary Figure 11. The single SNP analysis for BMI on Telomere, Supplementary Figure 12. The funnel plots for BMI on Telomere, Supplementary Figure 13. Scatterplot of MR analysis of BMI on Telomere, Supplementary Figure 14. The leave-one-out analysis for GrimAge on BMI, Supplementary Figure 15. The single SNP analysis for GrimAge on BMI, Supplementary Figure 16. The funnel plots for GrimAge on BMI [file 12944_2024_2042_MOESM3_ESM.zip › Supplementary figures 1-16/Supplementary Figure 14. The leave-one-out analysis for GrimAge on BMI.tif]

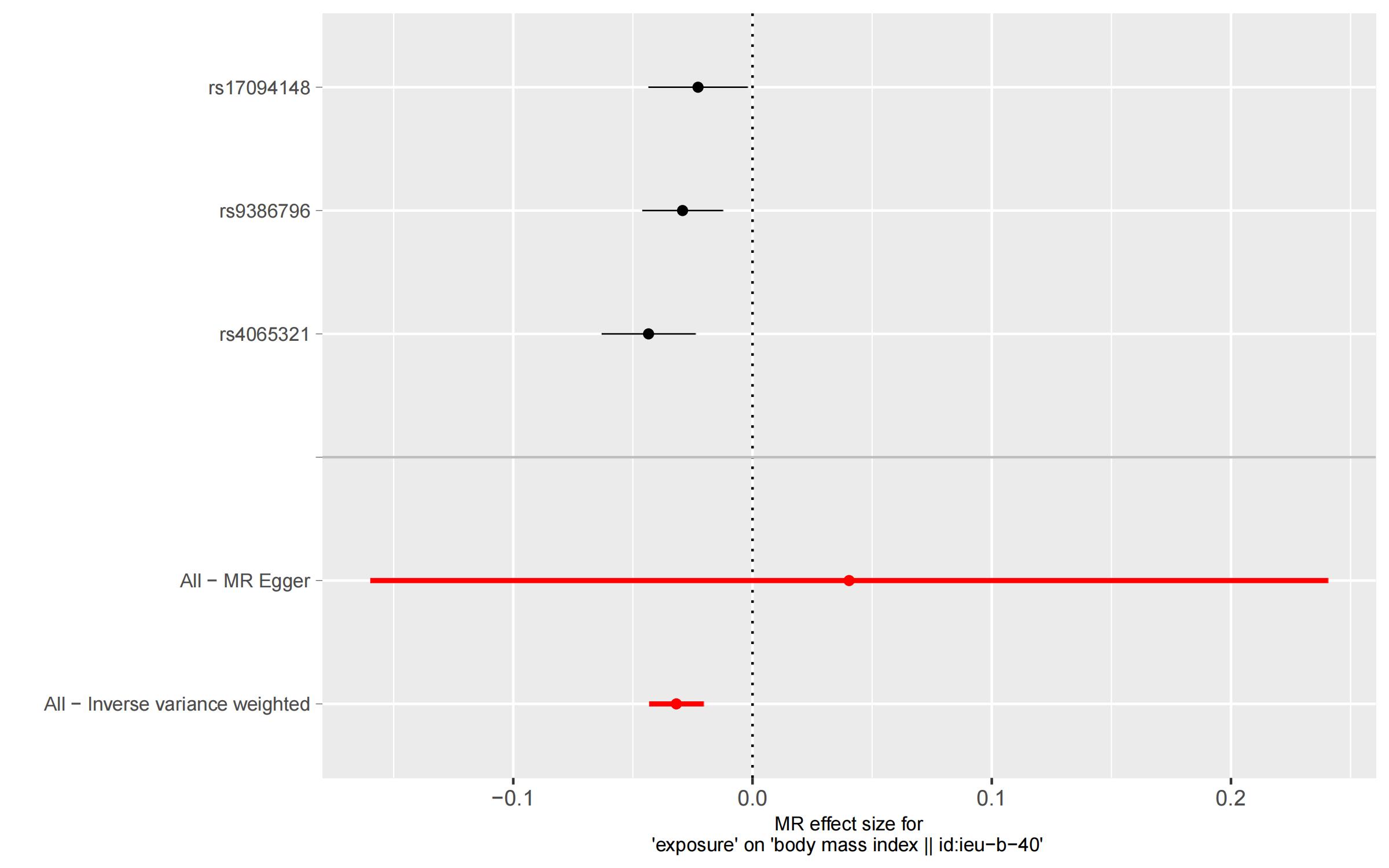

Supplement: Supplementary file 3 — Additional file 3:Supplementary Figure 1. Scatterplot of MR analysis of BMI on GrimAge, Supplementary Figure 2. Scatterplot of MR analysis of BMI on PhenoAge, Supplementary Figure 3. The leave-one-out analysis for BMI on GrimAge, Supplementary Figure 4. The leave-one-out analysis for BMI on PhenoAge, Supplementary Figure 5. The single SNP analysis for BMI on GrimAge, Supplementary Figure 6. The single SNP analysis for BMI on PhenoAge, Supplementary Figure 7. The funnel plots for BMI on GrimAge, Supplementary Figure 8. The funnel plots for BMI on PhenoAge, Supplementary Figure 9. Scatterplot of MR analysis of BMI on Telomere, Supplementary Figure 10. The leave-one-out analysis for BMI on Telomere, Supplementary Figure 11. The single SNP analysis for BMI on Telomere, Supplementary Figure 12. The funnel plots for BMI on Telomere, Supplementary Figure 13. Scatterplot of MR analysis of BMI on Telomere, Supplementary Figure 14. The leave-one-out analysis for GrimAge on BMI, Supplementary Figure 15. The single SNP analysis for GrimAge on BMI, Supplementary Figure 16. The funnel plots for GrimAge on BMI [file 12944_2024_2042_MOESM3_ESM.zip › Supplementary figures 1-16/Supplementary Figure 15. The single SNP analysis for GrimAge on BMI.tif]

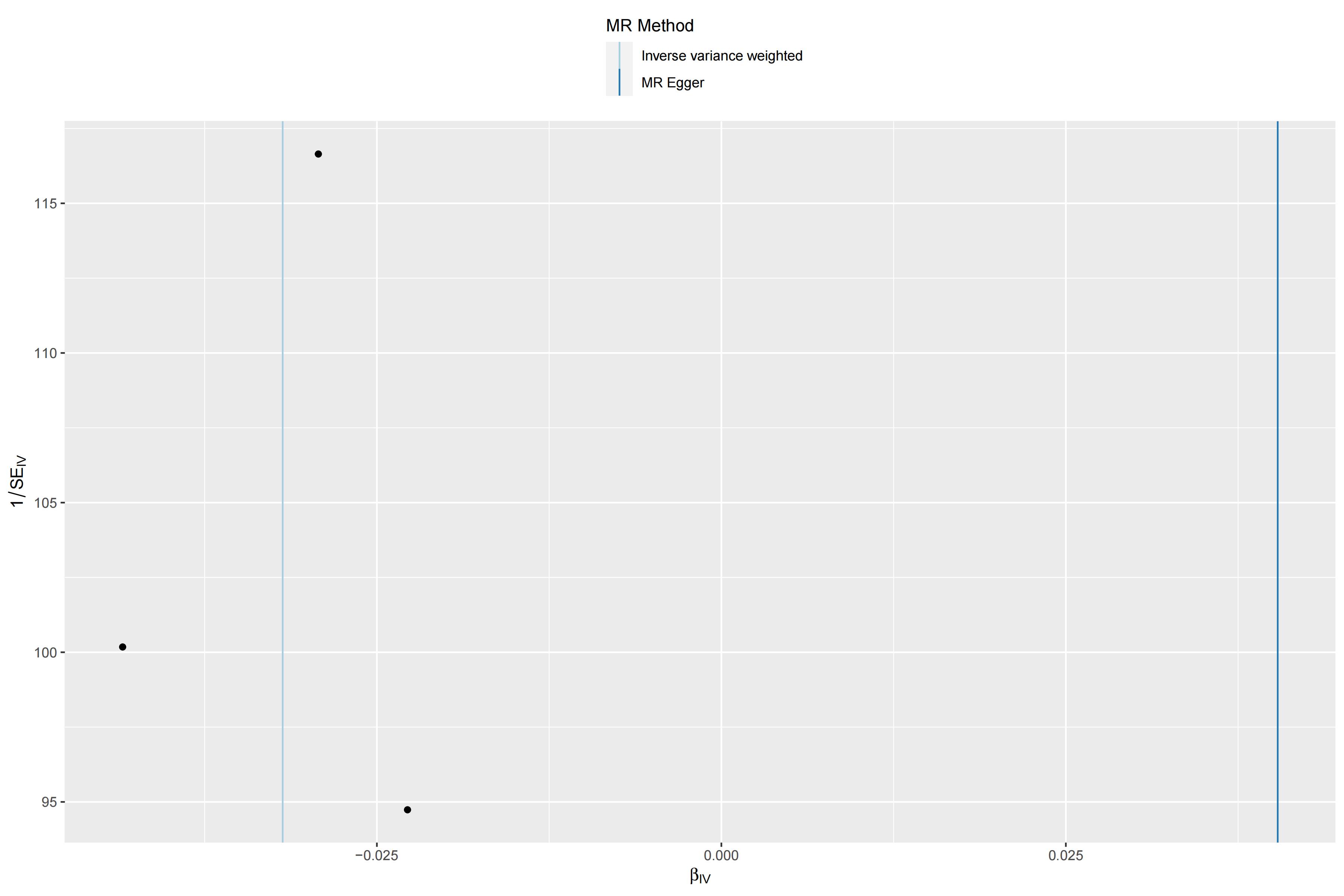

Supplement: Supplementary file 3 — Additional file 3:Supplementary Figure 1. Scatterplot of MR analysis of BMI on GrimAge, Supplementary Figure 2. Scatterplot of MR analysis of BMI on PhenoAge, Supplementary Figure 3. The leave-one-out analysis for BMI on GrimAge, Supplementary Figure 4. The leave-one-out analysis for BMI on PhenoAge, Supplementary Figure 5. The single SNP analysis for BMI on GrimAge, Supplementary Figure 6. The single SNP analysis for BMI on PhenoAge, Supplementary Figure 7. The funnel plots for BMI on GrimAge, Supplementary Figure 8. The funnel plots for BMI on PhenoAge, Supplementary Figure 9. Scatterplot of MR analysis of BMI on Telomere, Supplementary Figure 10. The leave-one-out analysis for BMI on Telomere, Supplementary Figure 11. The single SNP analysis for BMI on Telomere, Supplementary Figure 12. The funnel plots for BMI on Telomere, Supplementary Figure 13. Scatterplot of MR analysis of BMI on Telomere, Supplementary Figure 14. The leave-one-out analysis for GrimAge on BMI, Supplementary Figure 15. The single SNP analysis for GrimAge on BMI, Supplementary Figure 16. The funnel plots for GrimAge on BMI [file 12944_2024_2042_MOESM3_ESM.zip › Supplementary figures 1-16/Supplementary Figure 16. The funnel plots for GrimAge on BMI.tif]

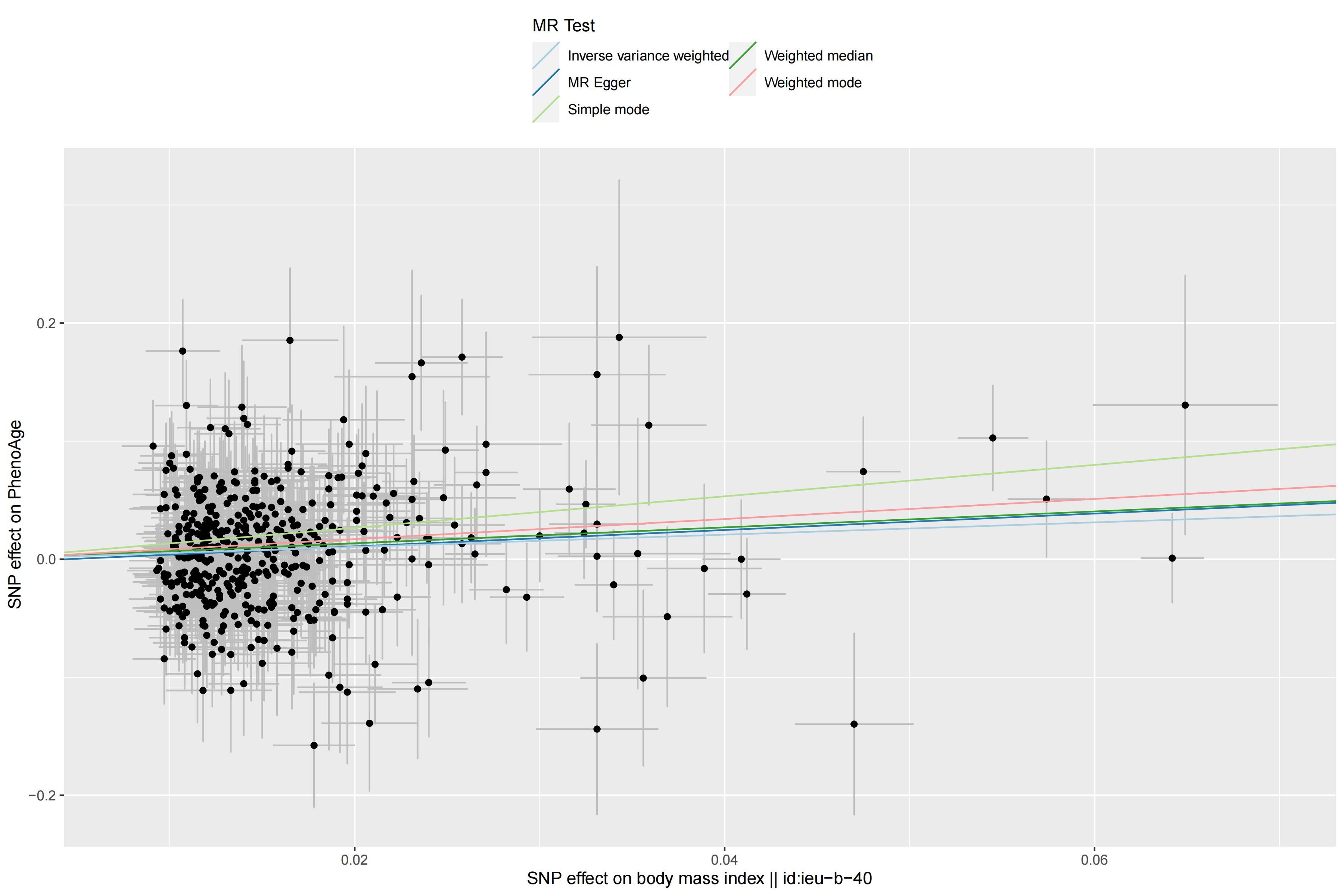

Supplement: Supplementary file 3 — Additional file 3:Supplementary Figure 1. Scatterplot of MR analysis of BMI on GrimAge, Supplementary Figure 2. Scatterplot of MR analysis of BMI on PhenoAge, Supplementary Figure 3. The leave-one-out analysis for BMI on GrimAge, Supplementary Figure 4. The leave-one-out analysis for BMI on PhenoAge, Supplementary Figure 5. The single SNP analysis for BMI on GrimAge, Supplementary Figure 6. The single SNP analysis for BMI on PhenoAge, Supplementary Figure 7. The funnel plots for BMI on GrimAge, Supplementary Figure 8. The funnel plots for BMI on PhenoAge, Supplementary Figure 9. Scatterplot of MR analysis of BMI on Telomere, Supplementary Figure 10. The leave-one-out analysis for BMI on Telomere, Supplementary Figure 11. The single SNP analysis for BMI on Telomere, Supplementary Figure 12. The funnel plots for BMI on Telomere, Supplementary Figure 13. Scatterplot of MR analysis of BMI on Telomere, Supplementary Figure 14. The leave-one-out analysis for GrimAge on BMI, Supplementary Figure 15. The single SNP analysis for GrimAge on BMI, Supplementary Figure 16. The funnel plots for GrimAge on BMI [file 12944_2024_2042_MOESM3_ESM.zip › Supplementary figures 1-16/Supplementary Figure 2. Scatterplot of MR analysis of BMI on PhenoAge.tif]

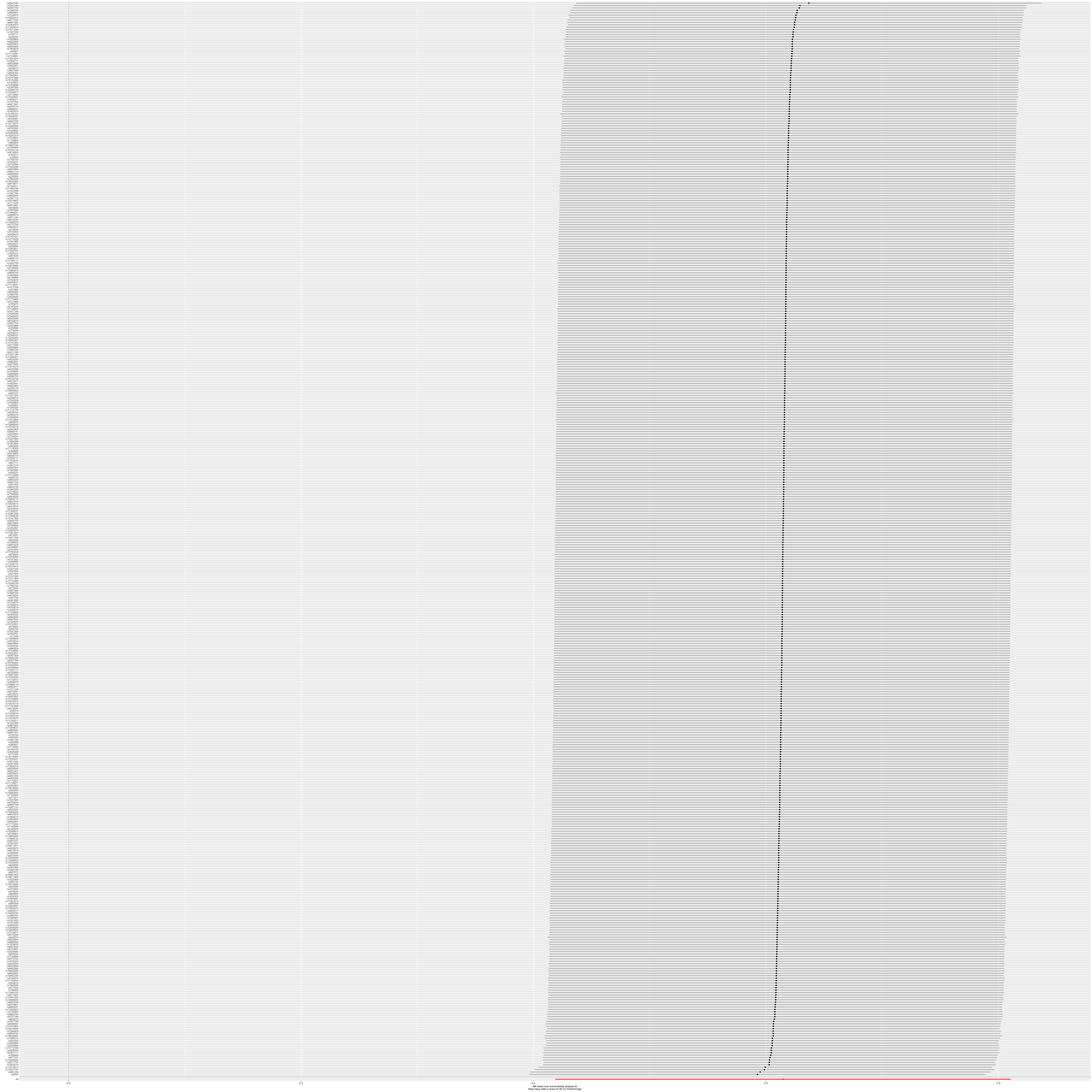

Supplement: Supplementary file 3 — Additional file 3:Supplementary Figure 1. Scatterplot of MR analysis of BMI on GrimAge, Supplementary Figure 2. Scatterplot of MR analysis of BMI on PhenoAge, Supplementary Figure 3. The leave-one-out analysis for BMI on GrimAge, Supplementary Figure 4. The leave-one-out analysis for BMI on PhenoAge, Supplementary Figure 5. The single SNP analysis for BMI on GrimAge, Supplementary Figure 6. The single SNP analysis for BMI on PhenoAge, Supplementary Figure 7. The funnel plots for BMI on GrimAge, Supplementary Figure 8. The funnel plots for BMI on PhenoAge, Supplementary Figure 9. Scatterplot of MR analysis of BMI on Telomere, Supplementary Figure 10. The leave-one-out analysis for BMI on Telomere, Supplementary Figure 11. The single SNP analysis for BMI on Telomere, Supplementary Figure 12. The funnel plots for BMI on Telomere, Supplementary Figure 13. Scatterplot of MR analysis of BMI on Telomere, Supplementary Figure 14. The leave-one-out analysis for GrimAge on BMI, Supplementary Figure 15. The single SNP analysis for GrimAge on BMI, Supplementary Figure 16. The funnel plots for GrimAge on BMI [file 12944_2024_2042_MOESM3_ESM.zip › Supplementary figures 1-16/Supplementary Figure 3. The leave-one-out analysis for BMI on GrimAge.tif]

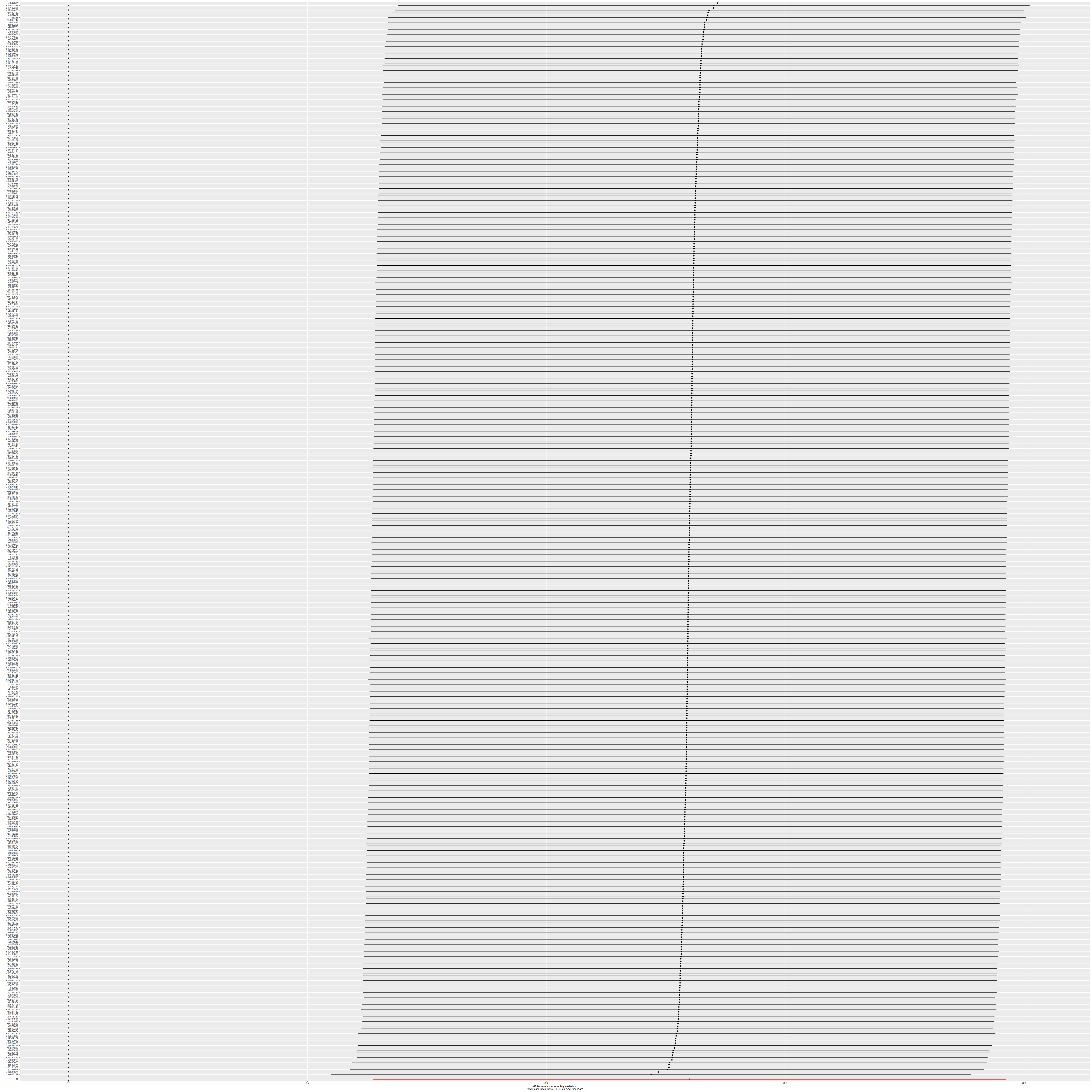

Supplement: Supplementary file 3 — Additional file 3:Supplementary Figure 1. Scatterplot of MR analysis of BMI on GrimAge, Supplementary Figure 2. Scatterplot of MR analysis of BMI on PhenoAge, Supplementary Figure 3. The leave-one-out analysis for BMI on GrimAge, Supplementary Figure 4. The leave-one-out analysis for BMI on PhenoAge, Supplementary Figure 5. The single SNP analysis for BMI on GrimAge, Supplementary Figure 6. The single SNP analysis for BMI on PhenoAge, Supplementary Figure 7. The funnel plots for BMI on GrimAge, Supplementary Figure 8. The funnel plots for BMI on PhenoAge, Supplementary Figure 9. Scatterplot of MR analysis of BMI on Telomere, Supplementary Figure 10. The leave-one-out analysis for BMI on Telomere, Supplementary Figure 11. The single SNP analysis for BMI on Telomere, Supplementary Figure 12. The funnel plots for BMI on Telomere, Supplementary Figure 13. Scatterplot of MR analysis of BMI on Telomere, Supplementary Figure 14. The leave-one-out analysis for GrimAge on BMI, Supplementary Figure 15. The single SNP analysis for GrimAge on BMI, Supplementary Figure 16. The funnel plots for GrimAge on BMI [file 12944_2024_2042_MOESM3_ESM.zip › Supplementary figures 1-16/Supplementary Figure 4. The leave-one-out analysis for BMI on PhenoAge.tif]

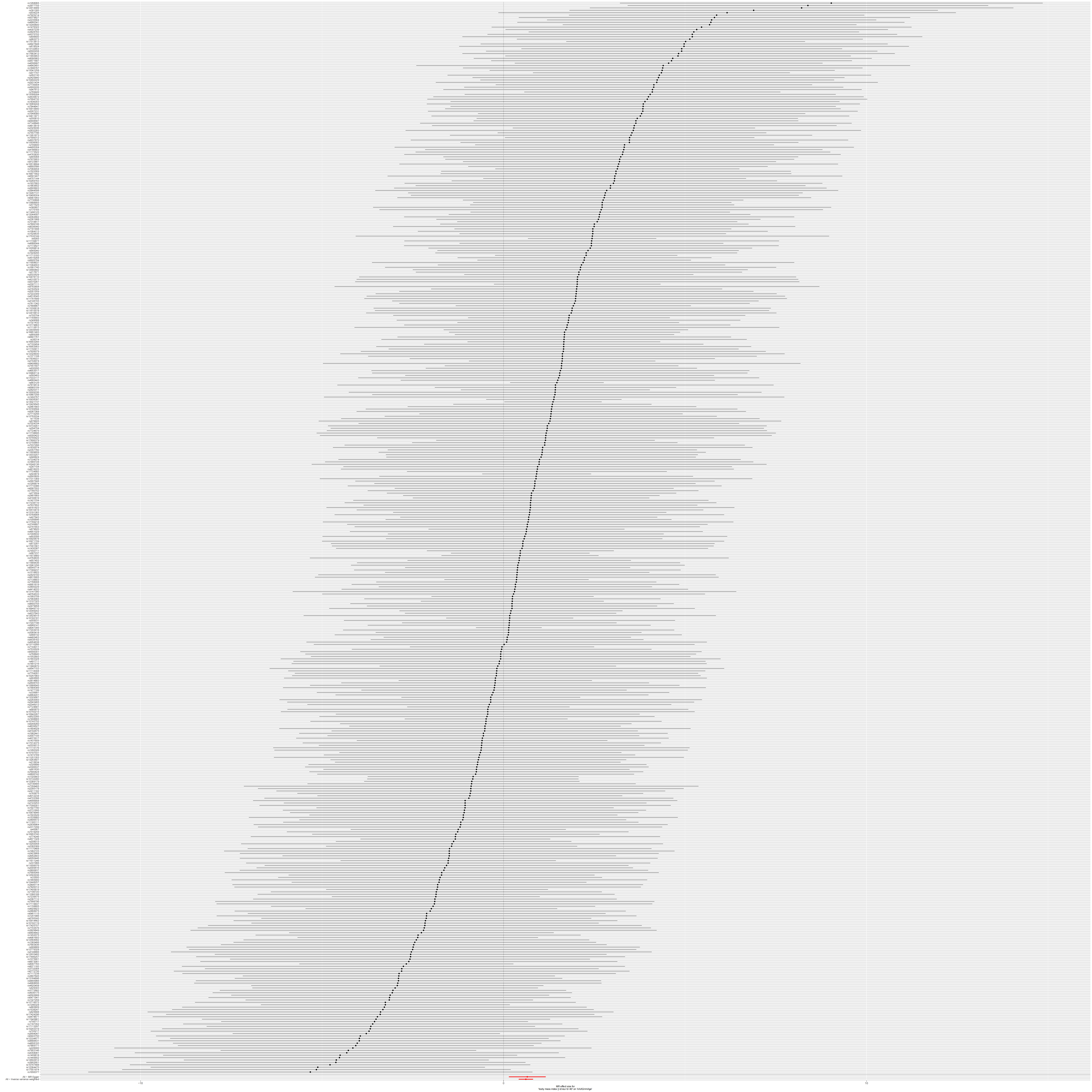

Supplement: Supplementary file 3 — Additional file 3:Supplementary Figure 1. Scatterplot of MR analysis of BMI on GrimAge, Supplementary Figure 2. Scatterplot of MR analysis of BMI on PhenoAge, Supplementary Figure 3. The leave-one-out analysis for BMI on GrimAge, Supplementary Figure 4. The leave-one-out analysis for BMI on PhenoAge, Supplementary Figure 5. The single SNP analysis for BMI on GrimAge, Supplementary Figure 6. The single SNP analysis for BMI on PhenoAge, Supplementary Figure 7. The funnel plots for BMI on GrimAge, Supplementary Figure 8. The funnel plots for BMI on PhenoAge, Supplementary Figure 9. Scatterplot of MR analysis of BMI on Telomere, Supplementary Figure 10. The leave-one-out analysis for BMI on Telomere, Supplementary Figure 11. The single SNP analysis for BMI on Telomere, Supplementary Figure 12. The funnel plots for BMI on Telomere, Supplementary Figure 13. Scatterplot of MR analysis of BMI on Telomere, Supplementary Figure 14. The leave-one-out analysis for GrimAge on BMI, Supplementary Figure 15. The single SNP analysis for GrimAge on BMI, Supplementary Figure 16. The funnel plots for GrimAge on BMI [file 12944_2024_2042_MOESM3_ESM.zip › Supplementary figures 1-16/Supplementary Figure 5. The single SNP analysis for BMI on GrimAge.tif]

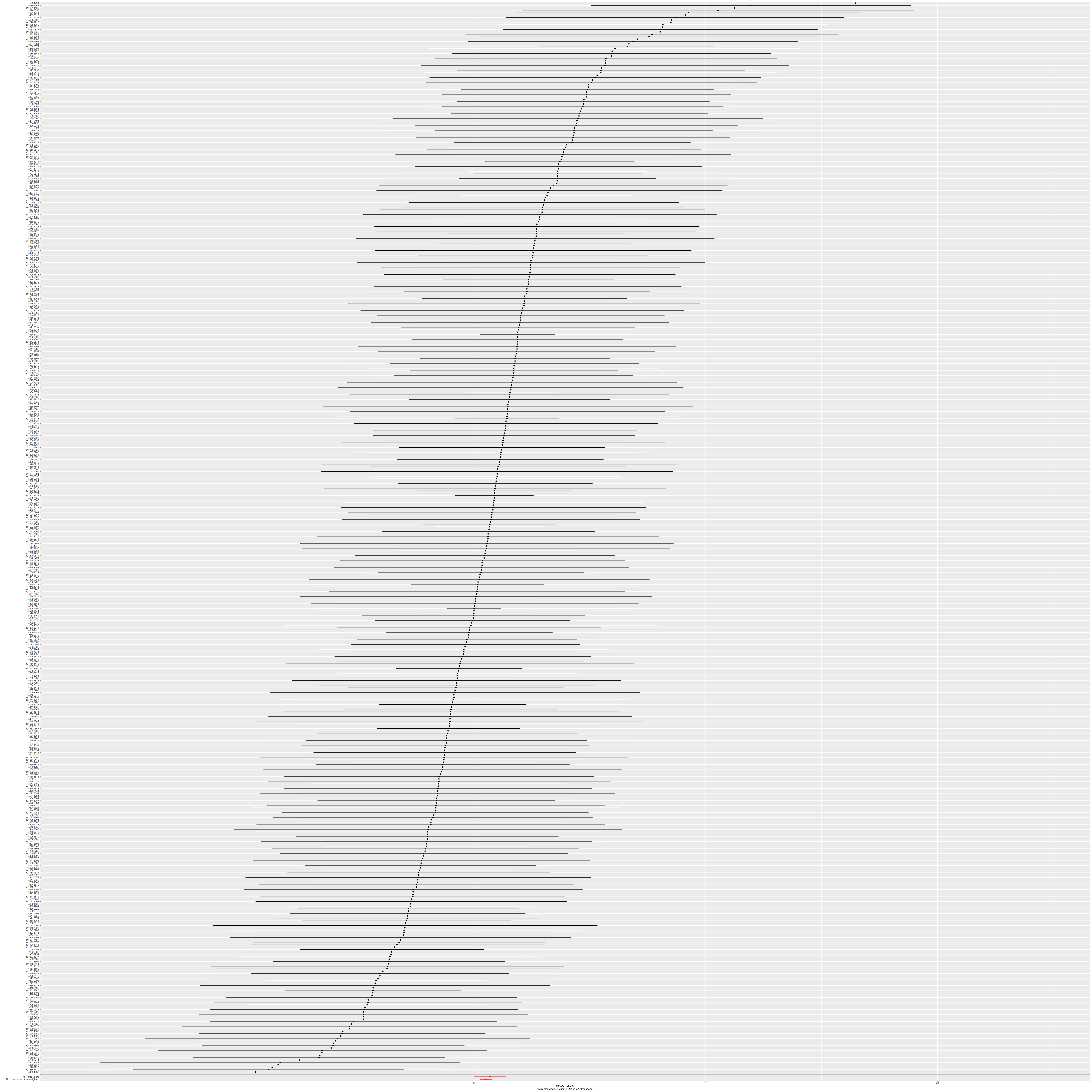

Supplement: Supplementary file 3 — Additional file 3:Supplementary Figure 1. Scatterplot of MR analysis of BMI on GrimAge, Supplementary Figure 2. Scatterplot of MR analysis of BMI on PhenoAge, Supplementary Figure 3. The leave-one-out analysis for BMI on GrimAge, Supplementary Figure 4. The leave-one-out analysis for BMI on PhenoAge, Supplementary Figure 5. The single SNP analysis for BMI on GrimAge, Supplementary Figure 6. The single SNP analysis for BMI on PhenoAge, Supplementary Figure 7. The funnel plots for BMI on GrimAge, Supplementary Figure 8. The funnel plots for BMI on PhenoAge, Supplementary Figure 9. Scatterplot of MR analysis of BMI on Telomere, Supplementary Figure 10. The leave-one-out analysis for BMI on Telomere, Supplementary Figure 11. The single SNP analysis for BMI on Telomere, Supplementary Figure 12. The funnel plots for BMI on Telomere, Supplementary Figure 13. Scatterplot of MR analysis of BMI on Telomere, Supplementary Figure 14. The leave-one-out analysis for GrimAge on BMI, Supplementary Figure 15. The single SNP analysis for GrimAge on BMI, Supplementary Figure 16. The funnel plots for GrimAge on BMI [file 12944_2024_2042_MOESM3_ESM.zip › Supplementary figures 1-16/Supplementary Figure 6. The single SNP analysis for BMI on PhenoAge.tif]

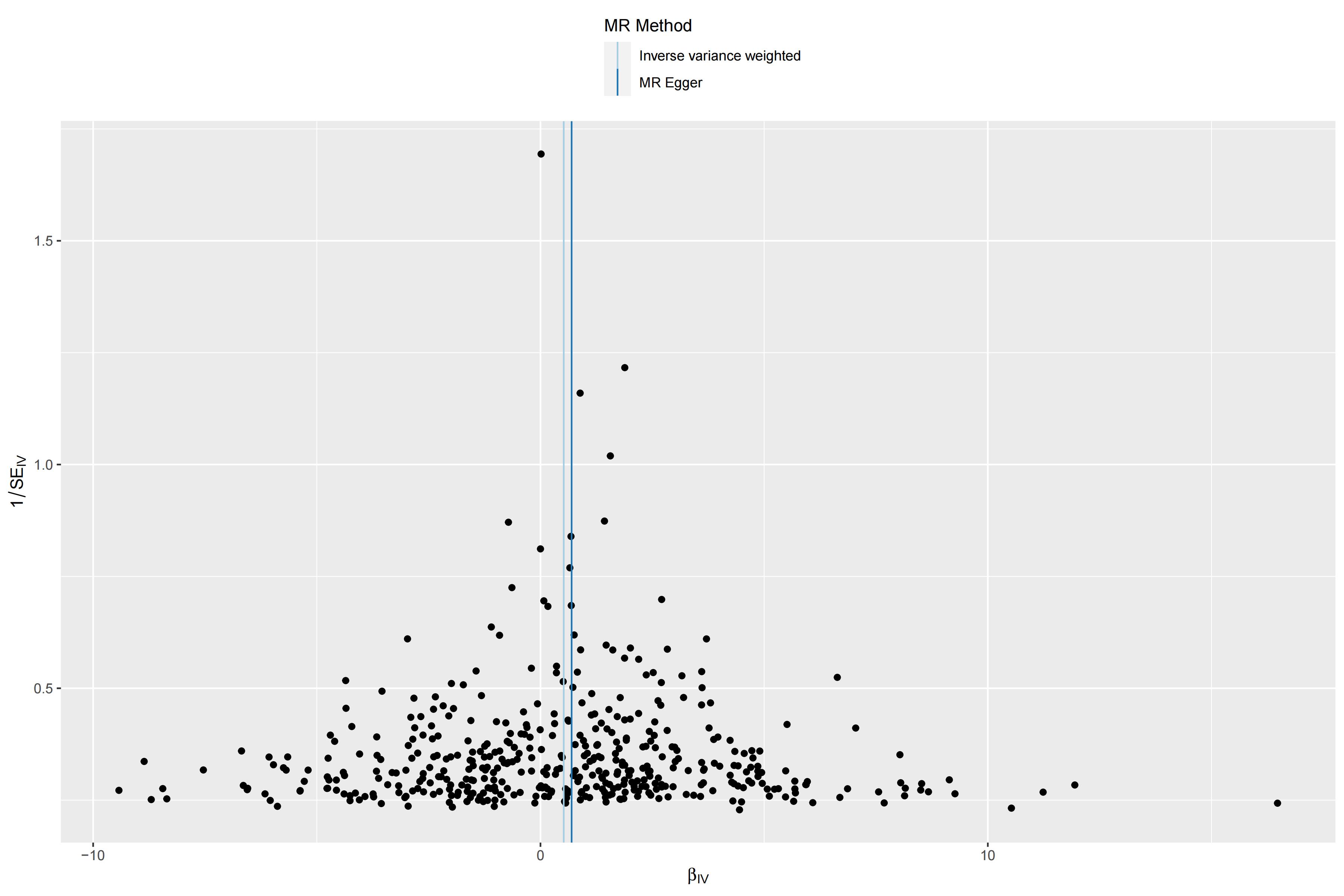

Supplement: Supplementary file 3 — Additional file 3:Supplementary Figure 1. Scatterplot of MR analysis of BMI on GrimAge, Supplementary Figure 2. Scatterplot of MR analysis of BMI on PhenoAge, Supplementary Figure 3. The leave-one-out analysis for BMI on GrimAge, Supplementary Figure 4. The leave-one-out analysis for BMI on PhenoAge, Supplementary Figure 5. The single SNP analysis for BMI on GrimAge, Supplementary Figure 6. The single SNP analysis for BMI on PhenoAge, Supplementary Figure 7. The funnel plots for BMI on GrimAge, Supplementary Figure 8. The funnel plots for BMI on PhenoAge, Supplementary Figure 9. Scatterplot of MR analysis of BMI on Telomere, Supplementary Figure 10. The leave-one-out analysis for BMI on Telomere, Supplementary Figure 11. The single SNP analysis for BMI on Telomere, Supplementary Figure 12. The funnel plots for BMI on Telomere, Supplementary Figure 13. Scatterplot of MR analysis of BMI on Telomere, Supplementary Figure 14. The leave-one-out analysis for GrimAge on BMI, Supplementary Figure 15. The single SNP analysis for GrimAge on BMI, Supplementary Figure 16. The funnel plots for GrimAge on BMI [file 12944_2024_2042_MOESM3_ESM.zip › Supplementary figures 1-16/Supplementary Figure 7. The funnel plots for BMI on GrimAge.tif]

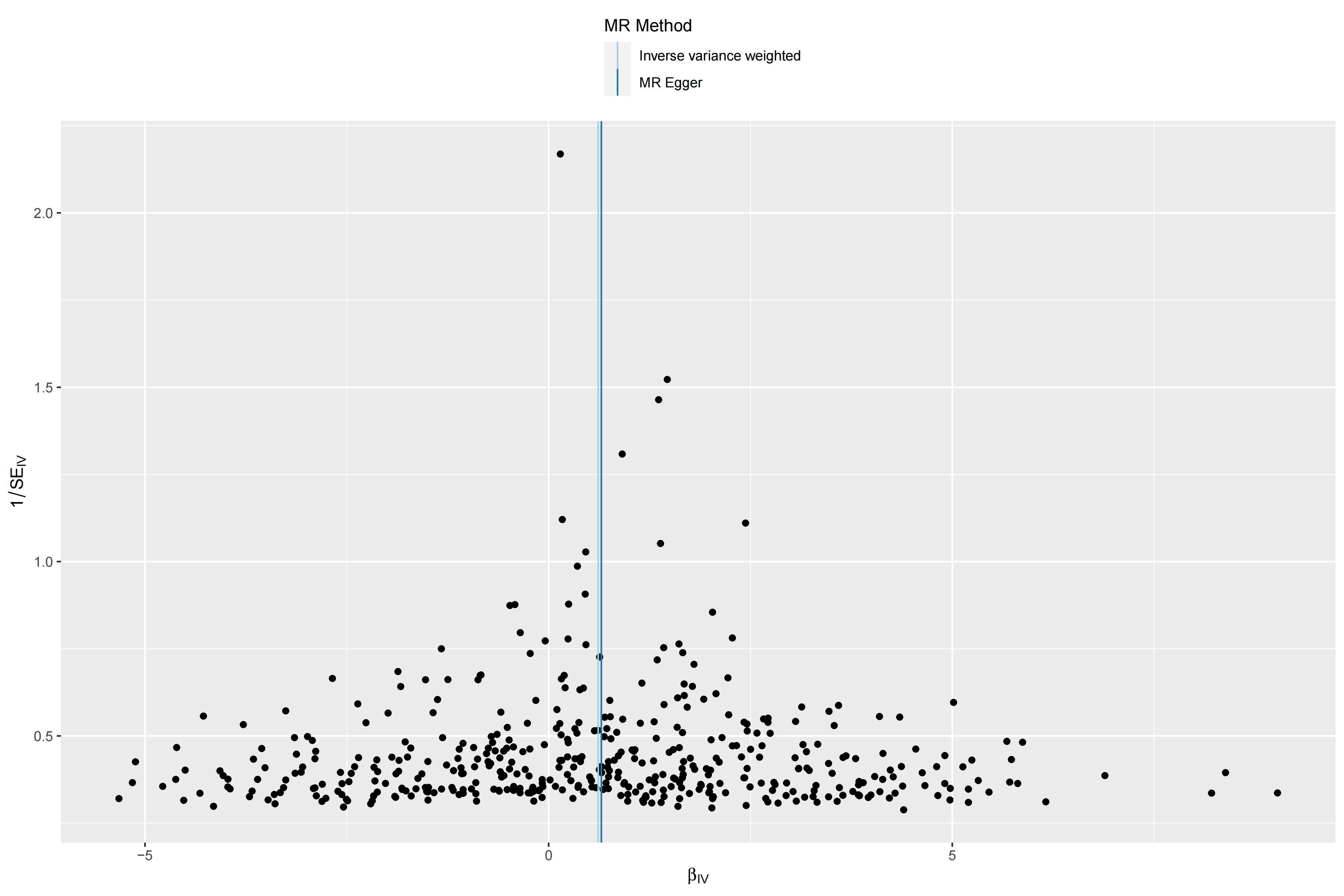

Supplement: Supplementary file 3 — Additional file 3:Supplementary Figure 1. Scatterplot of MR analysis of BMI on GrimAge, Supplementary Figure 2. Scatterplot of MR analysis of BMI on PhenoAge, Supplementary Figure 3. The leave-one-out analysis for BMI on GrimAge, Supplementary Figure 4. The leave-one-out analysis for BMI on PhenoAge, Supplementary Figure 5. The single SNP analysis for BMI on GrimAge, Supplementary Figure 6. The single SNP analysis for BMI on PhenoAge, Supplementary Figure 7. The funnel plots for BMI on GrimAge, Supplementary Figure 8. The funnel plots for BMI on PhenoAge, Supplementary Figure 9. Scatterplot of MR analysis of BMI on Telomere, Supplementary Figure 10. The leave-one-out analysis for BMI on Telomere, Supplementary Figure 11. The single SNP analysis for BMI on Telomere, Supplementary Figure 12. The funnel plots for BMI on Telomere, Supplementary Figure 13. Scatterplot of MR analysis of BMI on Telomere, Supplementary Figure 14. The leave-one-out analysis for GrimAge on BMI, Supplementary Figure 15. The single SNP analysis for GrimAge on BMI, Supplementary Figure 16. The funnel plots for GrimAge on BMI [file 12944_2024_2042_MOESM3_ESM.zip › Supplementary figures 1-16/Supplementary Figure 8. The funnel plots for BMI on PhenoAge.tif]

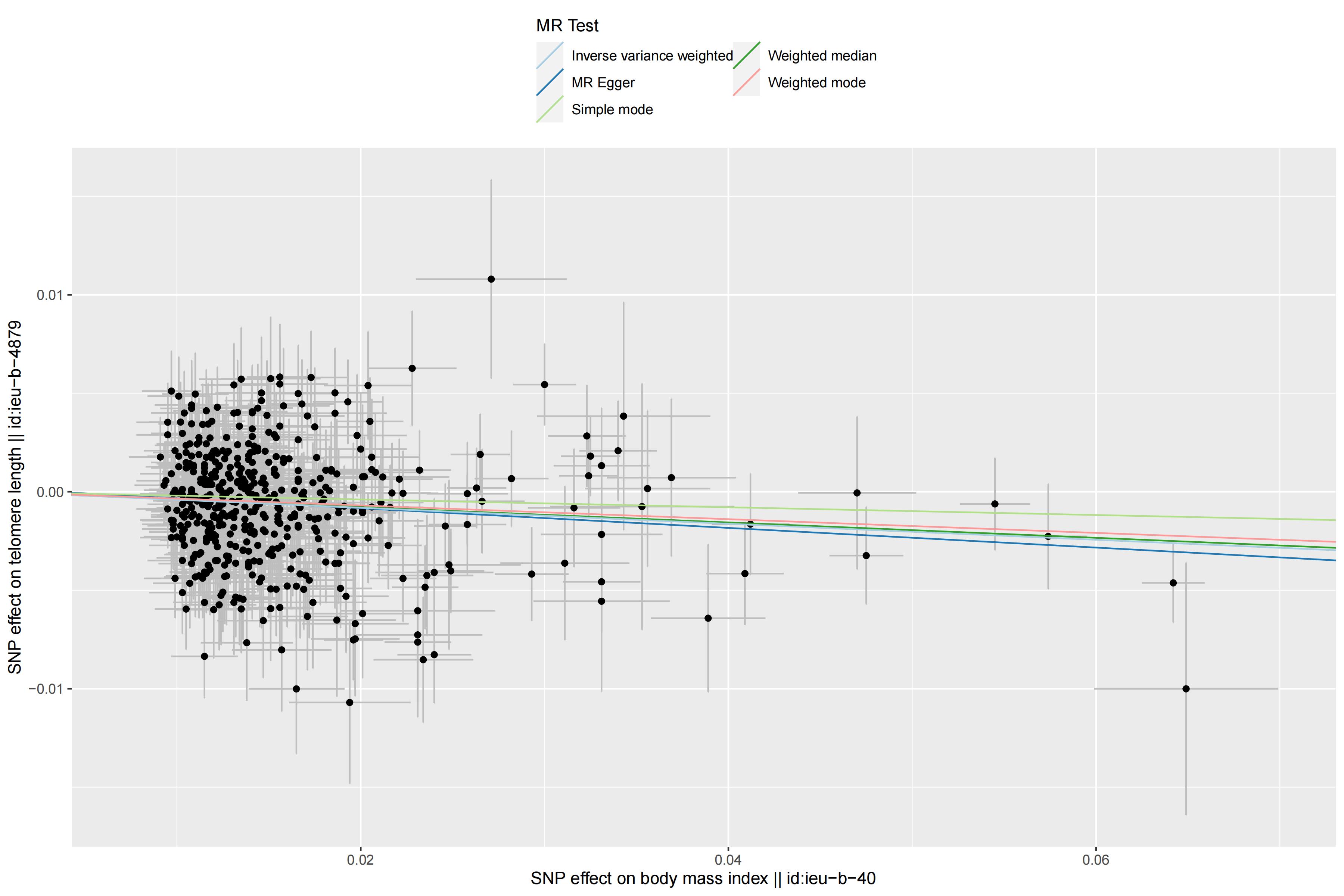

Supplement: Supplementary file 3 — Additional file 3:Supplementary Figure 1. Scatterplot of MR analysis of BMI on GrimAge, Supplementary Figure 2. Scatterplot of MR analysis of BMI on PhenoAge, Supplementary Figure 3. The leave-one-out analysis for BMI on GrimAge, Supplementary Figure 4. The leave-one-out analysis for BMI on PhenoAge, Supplementary Figure 5. The single SNP analysis for BMI on GrimAge, Supplementary Figure 6. The single SNP analysis for BMI on PhenoAge, Supplementary Figure 7. The funnel plots for BMI on GrimAge, Supplementary Figure 8. The funnel plots for BMI on PhenoAge, Supplementary Figure 9. Scatterplot of MR analysis of BMI on Telomere, Supplementary Figure 10. The leave-one-out analysis for BMI on Telomere, Supplementary Figure 11. The single SNP analysis for BMI on Telomere, Supplementary Figure 12. The funnel plots for BMI on Telomere, Supplementary Figure 13. Scatterplot of MR analysis of BMI on Telomere, Supplementary Figure 14. The leave-one-out analysis for GrimAge on BMI, Supplementary Figure 15. The single SNP analysis for GrimAge on BMI, Supplementary Figure 16. The funnel plots for GrimAge on BMI [file 12944_2024_2042_MOESM3_ESM.zip › Supplementary figures 1-16/Supplementary Figure 9. Scatterplot of MR analysis of BMI on Telomere.tif]
